# Integrated Information in Discrete Dynamical Systems: Motivation and Theoretical Framework

**DOI:** 10.1371/journal.pcbi.1000091

**Published:** 2008-06-13

**Authors:** David Balduzzi, Giulio Tononi

**Affiliations:** Department of Psychiatry, University of Wisconsin, Madison, Wisconsin, United States of America; Indiana University, United States of America

## Abstract

This paper introduces a time- and state-dependent measure of integrated information, *φ*, which captures the repertoire of causal states available to a system as a whole. Specifically, *φ* quantifies how much information is generated (uncertainty is reduced) when a system enters a particular state through causal interactions among its elements, above and beyond the information generated independently by its parts. Such mathematical characterization is motivated by the observation that integrated information captures two key phenomenological properties of consciousness: (i) there is a large repertoire of conscious experiences so that, when one particular experience occurs, it generates a large amount of information by ruling out all the others; and (ii) this information is integrated, in that each experience appears as a whole that cannot be decomposed into independent parts. This paper extends previous work on stationary systems and applies integrated information to discrete networks as a function of their dynamics and causal architecture. An analysis of basic examples indicates the following: (i) *φ* varies depending on the state entered by a network, being higher if active and inactive elements are balanced and lower if the network is inactive or hyperactive. (ii) *φ* varies for systems with identical or similar surface dynamics depending on the underlying causal architecture, being low for systems that merely copy or replay activity states. (iii) *φ* varies as a function of network architecture. High *φ* values can be obtained by architectures that conjoin functional specialization with functional integration. Strictly modular and homogeneous systems cannot generate high *φ* because the former lack integration, whereas the latter lack information. Feedforward and lattice architectures are capable of generating high *φ* but are inefficient. (iv) In Hopfield networks, *φ* is low for attractor states and neutral states, but increases if the networks are optimized to achieve tension between local and global interactions. These basic examples appear to match well against neurobiological evidence concerning the neural substrates of consciousness. More generally, *φ* appears to be a useful metric to characterize the capacity of any physical system to integrate information.

## Author Summary

We have suggested that consciousness has to do with a system's capacity to generate *integrated information*. This suggestion stems from considering two basic properties of consciousness: (i) each conscious experience generates a large amount of information, by ruling out alternative experiences; and (ii) the information is integrated, meaning that it cannot be decomposed into independent parts. We introduce a measure that quantifies how much integrated information is generated by a discrete dynamical system in the process of transitioning from one state to the next. The measure captures the information generated by the causal interactions among the elements of the system, above and beyond the information generated independently by its parts. We present numerical analyses of basic examples, which match well against neurobiological evidence concerning the neural substrates of consciousness. The framework establishes an observer-independent view of information by taking an intrinsic perspective on interactions.

## Introduction

Scientists and engineers are usually interested in how information can be transmitted or stored from the perspective of a user. However, it is just as important to consider information, in the classic sense of reduction of uncertainty, from the perspective of an autonomous system. How much information is generated when the system enters a particular state by virtue of causal interactions among its elements? And to what extent is the information generated by the system as a whole, as opposed to the information generated independently by its parts? Addressing these questions requires the development of a new framework that is based on the notion of integrated information [1],[2].

The need for such a framework is not merely academic. Indeed, it was initially motivated by one of the most baffling scientific problems – the generation of conscious experience by the brain. We know that certain regions of the brain, for example the thalamocortical system [3], are essential for consciousness, whereas other regions, such as the cerebellum, are not, though the cerebellum has even more neurons and is seemingly just as complicated. We also know that consciousness fades during sleep early in the night, although neurons in the thalamocortical system remain just as active as during quiet wakefulness. During generalized seizures neurons fire even more strongly and synchronously, yet consciousness is suspended or much reduced. Why is this the case? Specifically, what are the necessary and sufficient conditions for a physical system to generate experience? This problem – also known as the first problem of consciousness – is thought to be rather hard, as it is not easy to see how “subjective” experience could be squeezed out of a collection of physical elements.

The integrated information theory of consciousness represents an attempt to address the first problem of consciousness from first principles [4],[5]. The theory argues that consciousness is integrated information, starting from a phenomenological analysis. It proceeds by defining integrated information and suggesting how it can be measured for stationary systems. Finally, it shows that the integrated information perspective can provide a parsimonious account for several key empirical facts about the relationship between consciousness and the brain.

In the present work, our goal is to provide a definition and measure of integrated information for systems of discrete elements that evolve through time. This extension provides a framework for integrated information that is fully general and can be applied in principle to any kind of physical system. It also permits further predictions concerning the relationships between brain processes and consciousness. Finally, irrespective of the relevance for understanding consciousness, the notion of integrated information presented here may be useful for characterizing computational systems not merely as processors or stores of information, but as integrators of information.

It is useful to briefly examine the phenomenological observations that motivate the integrated information approach to consciousness. From a first-person perspective – the perspective of the system that is actually capable of generating subjective experience – two fundamental properties of consciousness are apparent: *i)* there is a large repertoire of conscious experiences. This means that, when one particular experience occurs, it generates a lot of information; *ii)* each experience is integrated, i.e. it appears as a whole that cannot be decomposed into independent parts [4],[5]. Since we tend to take consciousness for granted, these two properties are best understood by resorting to thought experiments: one involving a photodiode and the other a digital camera.

### 

#### Information

Consider the following: You are facing a blank screen that is alternately on and off, and you have been instructed to say “light” when the screen turns on and “dark” when it turns off. A photodiode – a very simple light-sensitive device – has also been placed in front of the screen, and is set up to beep when the screen emits light and to stay silent when it does not. The first problem of consciousness reduces to this: when you distinguish between the screen being on or off, you have the subjective experience of seeing light or dark. The photodiode can also distinguish between the screen being on or off, but presumably it does not have a subjective experience of light and dark. What is the key difference between you and the photodiode?

According to the theory, the difference has to do with how much information is generated when that distinction is made. Information is classically defined as reduction of uncertainty when a particular outcome occurs out of a repertoire of alternative outcomes: the more numerous the outcomes, the greater the reduction of uncertainty, and thus the information. When the blank screen turns off, the photodiode enters one of its two possible states and beeps, yielding 1 bit of information. However, when you see the blank screen turn off, the state you enter rules out a very large number of possible states. Imagine that, instead of turning homogeneously off, the screen were to display at random every frame from every movie that was ever produced. Without any effort, each of these frames would cause you to enter a different state and see a different image. This means that when you enter the particular state (“seeing pure darkness”) you rule out not just “seeing light,” but an extraordinarily large number of alternative possibilities. Whether or not you think of the bewildering number of alternatives (you won't and you can't), this corresponds to an extraordinary amount of information. Importantly, this information has nothing to do with how complicated the scene is – pure darkness or a busy city street – but only with the number of alternative outcomes.

#### Integration

While the ability to distinguish among a large number of states is a fundamental difference between you and the photodiode, by itself it is not enough to account for the presence of consciousness. To see why, consider an idealized megapixel digital camera, whose sensor chip is essentially a collection of a million photodiodes. Even if each photodiode in the sensor chip were just binary, the camera could distinguish among 2^1,000,000^ states, an immense number, corresponding to 1,000,000 bits of information. Indeed, the camera would enter a different state for every frame from every movie that was ever produced. Yet few would argue that the camera is conscious. What is the key difference between you and the camera?

According to the theory, the difference has to do with integrated information. An external observer may consider the camera chip as a single system with a repertoire of 2^1,000,000^ states. In reality, however, the chip is not an integrated entity: since its 1,000,000 photodiodes have no way to interact, the state of each photodiode is causally independent of that of the others: in reality, the chip is a collection of 1,000,000 independent photodiodes, each with a repertoire of 2 states. This is easy to prove: if the sensor chip were cut down into its individual photodiodes, the performance of the camera would not change at all. By contrast, your vast repertoire of conscious states truly belongs to an integrated system, since it cannot be subdivided into repertoires of states available to independent components. Thus, a conscious image is always experienced as an integrated whole: no matter how hard you try, you cannot experience the left half of the visual field of view independently of the right half, or colors independent of shapes. Underlying this unity of experience are causal interactions within your brain, which make the state of each element causally dependent on that of other elements. Indeed, unlike the camera, your brain's performance breaks down if its elements are disconnected. And so does consciousness: for example, splitting the brain in two along the corpus callosum prevents causal interactions between the two hemispheres and splits experience in two – the right half of the visual field is experienced independently of the left.

This phenomenological analysis suggests that, to generate consciousness, a physical system must have a large repertoire of available states (information) *and* it must be unified, i.e. it should not be decomposable into a collection of causally independent subsystems (integration). How can one establish the size of the repertoire of states available to a unified system?

Our goal is to provide a way to measure how much information is generated when a physical system enters one particular state out of a repertoire of possible states, but only to the extent that the information is generated by the system as a whole, above and beyond the information generated independently by its parts. Previous work [2],[4],[5] focused on neural systems modeled as stationary multidimensional Gaussian processes. This had the advantage that analytical results could be obtained, but suffered from the drawback that time and the changing dynamics of the systems were not taken into account. In this paper, we extend the theory to include time as a discrete variable. We apply the theory to simple examples, discrete systems of a dozen or fewer elements. Although these systems are too small to be considered at all realistic, we choose them to illustrate the relationship between integrated information and the anatomical connectivity, causal architecture, dynamics, and noise levels of the networks.

## Models

To evaluate how much integrated information is generated when a system enters a particular state, we consider simple systems composed of a few interacting elements. Though the present framework is meant to be general, it is convenient to think of neural elements that can be active (fire) or inactive and can communicate through directed connections.

Let *X* be a system consisting of *n* elements, which are taken to be abstract indivisible units. Each element is assumed to have a finite repertoire of outputs, with no accessible internal structure. In the examples below the repertoire of the elements will typically consist of two outputs: 0 or 1, corresponding to silence or firing. The internal states of the elements are irrelevant because it is only through outputs that an element can causally affect the rest of the system.

Elements are linked by connections to form a directed graph, specifying which source elements are capable of affecting which target elements. Each target element is endowed with a “mechanism” or rule through which it determines its next output based on the inputs it receives. These mechanisms are assumed to be elementary, for example *AND, XOR*; they can also be probabilistic.

Time is assumed to pass in discrete instants, which could correspond to milliseconds for example. We use the word state to refer to the total output of a given subset of a discrete system at a given instant in time. Finally, the elements are memoryless, meaning they are modeled as first order Markov processes: the output of an element at time *t* depends only on the inputs at time *t*−1. In future work we will extend the framework to include elements with memory and explain how the natural time frame over which a system generates integrated information is specified.

### 

#### Notation

We refer to systems and subsets of systems by capital letters: *X*, *S* and so forth. Uppercase letters with subscripts (*X*_0_, *S*_0_) denote probability distributions of perturbations that are physically imposed on the outputs of a subset at a given time, e.g. at *t* = 0. Lowercase letters with subscripts (*x*_1_, *s*_1_) denote events: the actual output of the subset in question at a particular time, e.g. at *t* = 1.

### Information

First, we need to evaluate how much information is generated by a system when it enters a particular state, *x*_1_, out of its repertoire (a repertoire is a probability distribution on the set of output states of a system). The information generated should be a function of how large the repertoire of possible states is, and how much uncertainty about the repertoire is reduced by entering state *x*_1_. Also, the reduction of uncertainty must be produced by interactions among the elements of the system acting through their causal mechanisms, which is why we call it *effective information*.

**Figure 1:**
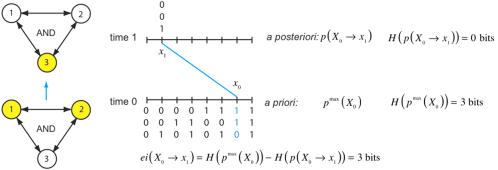
Effective information generated by entering a particular state. A system of three connected *AND*-gates transitions from state *x*_0_ = 110 at time zero to *x*_1_ = 001 at time one. The *a priori* repertoire is the uniform distribution on the 8 possible outputs of the elements of the system. The causal architecture of the system specifies that state 110 is the unique cause of *x*_1_, so the *a posteriori* repertoire (shown in cyan) assigns probability 1 to state 110 and 0 to all other states. Effective information generated by the system transitioning to *x*_1_ is 3 bits.

Let us first consider an isolated system, as in Figure 1. The system consists of three *AND*-gates and transitions from state *x*_0_ = 110 at time zero to state *x*_1_ = 001 at time one. How much effective information does the system generate? To answer the question we need to precisely describe: *i)* the alternative states available to the system (the *a priori* repertoire); *ii)* those states that the architecture of the system specifies as causes of *x*_1_ (the *a posteriori* repertoire). Effective information captures the information generated by the system by measuring the difference between these two repertoires.

***Effective information*** is defined as the entropy of the *a posteriori* repertoire relative to the *a priori* repertoire, which we write as:
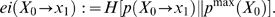
(1A)


***The a priori repertoire*** is the probability distribution on the set of possible outputs of the elements considered independently, with each output equally likely. This repertoire includes all possible states of the system prior to considering the effects of its causal architecture and the fact that it entered state *x*_1_. This distribution is imposed onto the system, i.e. we perform a perturbation in the sense of [6]. The *a priori* repertoire coincides with the maximum entropy (maxent) distribution on the states of the system; we denote it by *p* ^max^(*X*_0_). No perturbation can be ruled out *a priori*, since it is only by passing a state through the mechanism that the system generates information. The maximum entropy distribution formalizes the notion of complete ignorance [7]. In Figure 1 the *a priori* repertoire distribution assigns equal probability to each of the 2^3^ = 8 possible outputs of the system.

***The a posteriori repertoire*** *p*(*X*_0_ → *x*_1_) is the repertoire of states that could have led to *x*_1_ through causal interactions. We determine the *a posteriori* repertoire by forcibly intervening in the system and imposing each state in the *a priori* repertoire, thus we implement a perturbational approach [1],[2],[4],[6]; see also [8],[9] which apply perturbations to measure the average interaction between subsets for general distributions. Considering each *a priori* perturbation in turn we find that some perturbations could have caused (led to) *x*_1_ and others not (either deterministically or with a certain probability). The *a posteriori* repertoire is formally captured by Bayes' rule, which keeps track of which perturbations cause (lead to) the given effect (see Text S1, section 3). In Figure 1 *x*_0_ is the unique perturbation that causes *x*_1_, so the *a posteriori* repertoire assigns weight 1 to *x*_0_ and weight 0 to all other perturbations.

***Relative entropy*** (also known as Kullback-Leibler divergence, see Text S1, section 1) is the uncertainty reduction provided by an *a posteriori* repertoire with respect to an *a priori* repertoire. It is always non-negative, and is zero if and only if the repertoires are identical. In our case the information is generated by the system when, through causal interactions among its elements, it enters state *x*_1_ and thereby specifies an *a posteriori* distribution with respect to an *a priori* distribution. By comparing the *a priori* and *a posteriori* repertoires effective information measures those “differences that make a difference” [10].

Given that the second term is a maximum entropy distribution, Equation 1A can be more simply written as a difference of entropies, so that
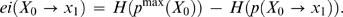
(1B)


Here *H*(*p*(−))is the entropy of probability distribution *p*. Entropy of the *a priori* repertoire *n* bits in a system of *n* binary elements. The second term is the entropy of the *a posteriori* repertoire, and lies between 0 and *n* bits depending on the state *x*_1_ and the architecture of the system. It follows that a system of *n* binary elements generates at most *n* bits of information.

In Figure 1 the entropy of the *a priori* repertoire is 3 bits and that of the *a posteriori* is 0 bits, so 3 bits of effective information are generated by the system when it enters *x*_1_: one out of eight perturbations is specified by the system as a cause of its current state, and the other 7 perturbations are ruled out, thus reducing uncertainty (generating information).

**Figure 2:**
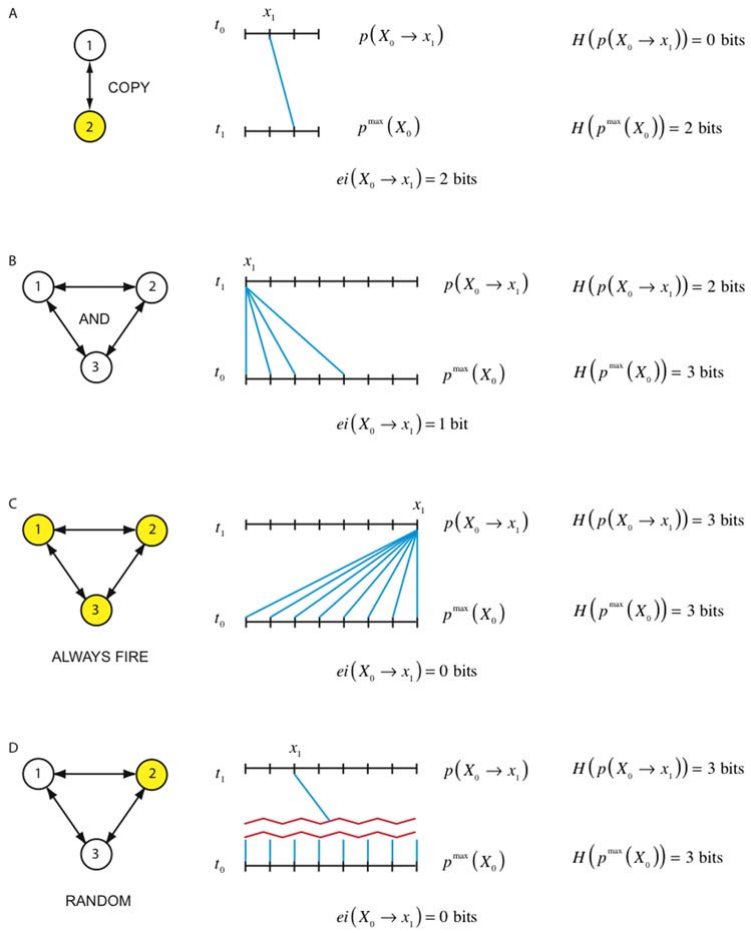
Effective information: a few examples. Each panel depicts a different system, which has entered a particular state. The *a priori* and *a posteriori* repertoires are shown and effective information is measured. (A) is a simple system of two elements that copy each other's previous outputs (a couple). Effective information is 2 bits, less than for the system in Figure 1 since the repertoire of outputs is smaller. (B) shows the *AND*-gate system of Figure 1 entering the state 000. This state is less informative than 001 since the *a posteriori* repertoire specified by the system includes four perturbations; effective information is reduced to 1 bit. The systems in (C) and (D) generate no effective information. In (C) the elements always fire regardless of their inputs, corresponding to an inescapable fixed point. In (D) the elements fire or are silent at random, so that the prior state is irrelevant. In both cases the *a posteriori* repertoire is the maximum entropy distribution since no alternatives have been ruled out, so effective information is zero.

In Figure 2, we show that effective information depends both on the size of the repertoire and on how much uncertainty is reduced by the mechanisms of the system. Figure 2A depicts a system of two elements. The *a priori* repertoire is smaller than in Figure 1, and effective information is reduced to 2 bits. Figure 2B shows the *AND*-gate system entering state *x*_1_ = 000. In this case the *a posteriori* repertoire specified by the system contains four perturbations that cannot be distinguished by its causal architecture, since each of the four perturbations leads to 000. Fewer alternatives from the *a priori* repertoire are ruled out, so effective information is 1 bit.

Finally, Figure 2C and 2D illustrate two systems that generate no effective information. In Figure 2C the elements fire no matter how the system is perturbed, so the system always enters state *x*_1_ = 111. The process of entering *x*_1_ does not rule out any alternative states, so the *a posteriori* repertoire coincides with the *a priori* repertoire and effective information is zero. In Figure 2D the elements fire or not with 50% probability no matter how the system is perturbed. In other words, the behavior of the system is completely dominated by noise. Again, the process of entering *x*_1_ does not rule out any alternative states, so the *a posteriori* repertoire coincides with the *a priori* repertoire and effective information is zero.

#### Effective information in systems that are not isolated

Up to now we have exclusively considered isolated systems. Suppose we embed *X* in some larger system *W* that forms the “world” of *X*. Inputs from the environment, *E* = *W* \ *X*, to *X* cannot be accounted for by *X* internally. From *X*'s point of view they are a source of extrinsic noise, since the information generated *by* the system must be due to causal interactions *within* the system. In general, to compute effective information one should average over all possible external inputs with the maximum entropy distribution (see Text S1, section 3, for details).

### Integrated information

Next, we must evaluate how much information is generated by a system above and beyond what can be accounted for by its parts acting independently.

**Figure 3:**
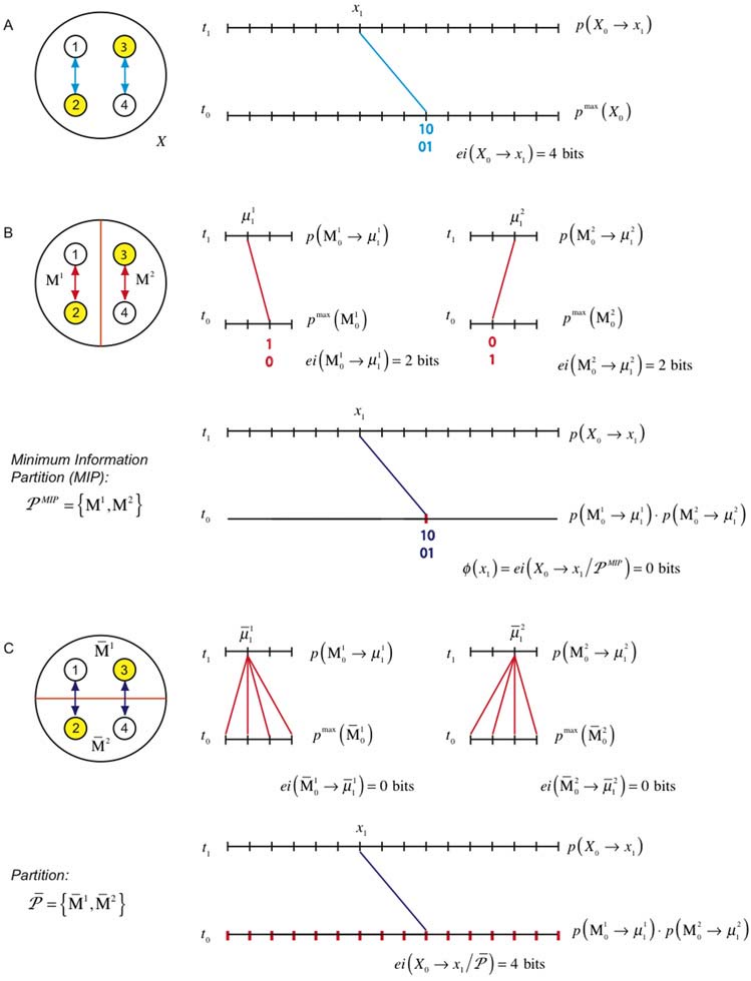
Integrated information for a system of two disjoint couples. The panels analyze the same system of two disjoint couples from three different perspectives. The interactions in the system are displayed in cyan. Those interactions that occur within a part are shown in red, and those between parts are in dark blue. (A) computes effective information for the entire system *X*, finding it to be 4 bits. (B) computes effective information generated by each of the couples independently and then computes integrated information *φ*(*x*_1_), finding it to be 0 bits since the two couples do not interact. Notice that the combined *a posteriori* repertoire of the parts coincides with the *a posteriori* repertoire of the system; the parts account for all the interactions within *X*. (C) considers a partition of the system other than the minimum information partition. Since 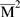 is not isolated it cannot account for the effect of interactions with 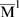 internally; they are treated as extrinsic noise and result in 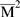 specifying a maximum entropy *a posteriori* repertoire. Effective information generated across the partition is 4 bits.

Consider Figure 3A. Effective information *ei*(*X*_0_ → *x*_1_) generated by the system, considered as a single entity, is 4 bits. In this case, however, it is clear that the two couples do not constitute a single entity at all: since there are no causal interactions between them, each of the disjoint couples generates 2 bits of information independently (Figure 3B). Effective information tells us how much information is generated without taking into account the extent to which the information is *integrated*. What we need to know, instead, is how much information is generated by the system as a whole, over and above the information generated independently by its parts, that is, we need to measure *integrated information*.

***Integrated information*** *φ* (I for information and O for integration) is defined as the entropy of the *a posteriori* repertoire of the system relative to the combined *a posteriori* repertoires of the parts:
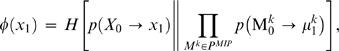
(2A)where M and *μ* stand for parts, and *P^MIP^* is the minimum information partition, which represents the natural decomposition of the system into parts.

**Figure 4:**
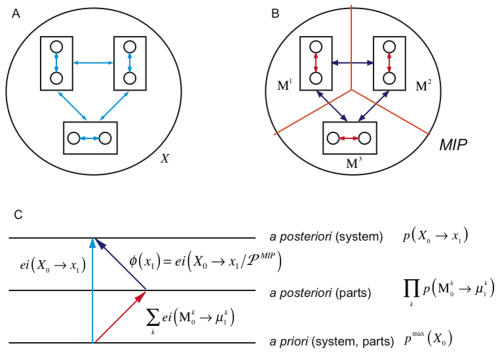
Effective information generated across the minimum information partition. (A) depicts the interactions within the system that are quantified by effective information of the entire system. (B) disentangles the interactions, showing interactions within parts in red, and interactions between parts in dark blue. (C) is a schematic of the relationship between the repertoires specified by the system and the parts. Effective information, represented by the arrows, is the entropy of a lower repertoire relative to an upper one. *φ*(*x*_1_) is the entropy of the *a posteriori* repertoire of the system relative to the combined *a posteriori* repertoire of the minimal parts.

***The a posteriori repertoires of the parts*** are found by considering each part as a system in its own right (averaging over inputs from other parts and extrinsic to the system, Figure 4). Each part has an *a priori* repertoire, given by the maximum entropy distribution. The product of the *a priori* repertoires of the parts is the same as the *a priori* repertoire of the system, since the elements are treated independently in both cases. The *a posteriori* repertoire 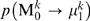 of each part M*^k^* is specified (as for the whole, *X*, in the previous section) by its causal architecture and current state 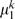, after averaging over external inputs. Thus the rest of the system is treated as a source of extrinsic noise by each part. The effective information generated independently by the parts, shown in red in Figure 4, is the sum of the entropies of their *a priori* repertoires relative to their *a posteriori* repertoires.

Integrated information, shown in dark blue, measures the information generated by the system through causal interactions among its elements (its *a posteriori* repertoire) with respect to (over and above) the information generated independently by its parts (their combined *a posteriori* repertoires). In particular, integrated information is zero if and only if the system can be decomposed into a collection of independent parts. Thus, *φ*(*x*_1_) of a system captures how much “the whole is more than the sum (or rather the product) of its parts.”

To exemplify, consider again the system of Figure 3, where the natural decomposition into parts is given by the subsets M^1^ and M^2^, as shown in Figure 3B. The *a posteriori* repertoire 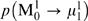 specifies perturbation 10. Similarly the *a posteriori* repertoire of M^2^ specifies perturbation 01. The combined *a posteriori* repertoire of the parts specifies perturbation 1001 (red notch), coinciding with the *a posteriori* perturbation specified by the entire system. No alternatives are ruled out by the system as a whole, so integrated information is
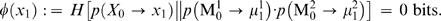



The system generates no information as a whole, over and above that generated by its parts.

Of note, a related measure is stochastic interaction [11], which quantifies the average interactions between subsets of a system. Briefly, our approach is distinguished by comparing the whole to the parts, rather than the parts to one another; see Text S1, section 8, for detailed discussion and technical motivation.

#### The minimum information partition

In the case of the two couples the natural decomposition of the system into parts is captured by partition *P^MIP^*. Considering other partitions, for example partition 
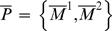
 in Figure 3C, would miss the obvious decomposition of the system into independent parts and lead to erroneous estimates of integrated information. This example suggests that, for any system, we need to find the informational “weakest link”, i.e. the decomposition into those parts that are most independent (least integrated). This weakest link is given by the minimum information partition *P^MIP^*, which can be found by searching over all partitions of the system after appropriate normalization.

To do so, let us define the *effective information across an arbitrary partition* 
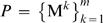
 as
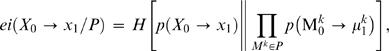
where the parts are mutually disjoint and collectively pave the system. A special case to consider is the total partition *P* = {*X*}. Since the part is the entire system, the *a posteriori* repertoire of the part equals that of the system, so if we apply Equation (2A) to the total partition we always obtain zero. Thus we define effective information across the total partition to be effective information generated by the entire system, as in Equation 1A. For a system containing no elements with self-connections effective information generated by the system (across the total partition) and effective information across the partition into individual elements coincide.

#### Normalization

Normalization is necessary because effective information across an asymmetric bipartition where one part contains a single element and the second part contains the rest will typically be less than across a symmetric partition into two parts of equal size. Similarly, effective information across partitions into many parts tends to be higher than across partitions into few parts. To fairly compare different partitions we therefore introduce the normalization:
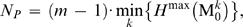
where *m* is the number of parts in the partition. The normalization is the size of the smallest *a priori* repertoire of a part multiplied by the number of other parts. In particular, for a partition into two parts *N_P_* is the size of the smaller *a priori* repertoire. The normalization for the total partition is *N_P_* = *H* ^max^(*X*_0_).

The *minimum information partition (MIP)* can then be defined as the partition for which normalized effective information is a minimum:
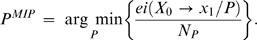



If there is more than one partition that attains the minimum normalized value, we select those partitions that generate the lowest un-normalized quantity of effective information to be the minimum information partition(s). Once the minimum information partition has been found, integrated information can be simply expressed as
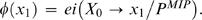
(2B)


#### Integrated information is bounded

For a discrete system composed of *n* binary elements *φ*(*x*_1_)≤*n* bits. This follows since the normalization is largest for the total partition, and for this partition effective information is 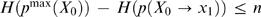 bits.

### Complexes

For any given system *X*, we are now in a position to identify those subsets that are capable of integrating information, the complexes. A subset *S* of *X* forms a *complex* when it enters state *s*_1_ if *φ*(*s*_1_)>0 and *S* is not contained in some larger set with strictly higher *φ*. A complex whose subsets have strictly lower *φ* is called a *main complex*. For instance, the complex in a given system with the maximum value of *φ* necessarily forms a main complex.
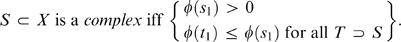
(3A)


In addition,
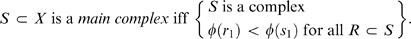
(3B)


At each instant in time any system of elements can be decomposed into its constituent complexes, which form its fundamental units. Indeed, only a complex can be properly considered to form a single entity. For a complex, and only for a complex, it is meaningful to say that, when it enters a particular state out of its repertoire, it generates an amount of integrated information corresponding to its *φ* value.

#### Decomposing a system into complexes

**Figure 5:**
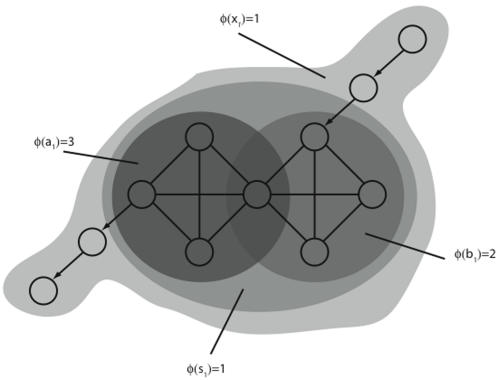
Decomposing systems into overlapping complexes. In this example elements are parity gates: they fire if they receive an odd number of spikes. Links without arrows are bidirectional. The system is decomposed into three of its complexes, shown in shades of gray. Observe that: *i)* complexes can overlap; *ii)* a complex can interact causally with elements not part of it; *iii)* groups of elements with identical architectures generate different amounts of integrated information, depending on their ports-in and ports-out (compare subset *A*, the dark gray filled-in circle, with subset *B*, the right-hand circle).

Figure 5 shows how a system *X* can be analyzed to find its constituent complexes, shown in shades of gray. From the figure, we see that complexes have the following properties: *i)* the same element can belong to more than one complex, and complexes can overlap; in particular, a smaller complex of high *φ* (main complex) may be contained within a larger complex of low *φ*; *ii)* a complex can be causally connected to elements that are not part of it (the input and output elements of a complex are called ports-in and ports-out, respectively); *iii)* groups of elements with identical causal architectures can generate different amounts of integrated information depending on their ports-in and ports-out (subsets *A* and *B* in Figure 5).

#### Elements independently driven by a complex do not generate integrated information

**Figure 6:**
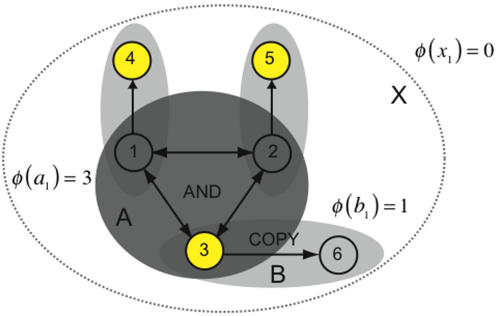
Elements driven by a complex do not contribute to integrated information. The system is constructed using the *AND*-gate system of Figure 1, with the addition of three elements copying the inner triple. The *AND*-triple forms a main complex, as do the couples. However, the entire system generates no integrated information and does *not* form a complex, since *X* generates no information over and above that generated by subset *A*.

Figure 6 shows a system of interacting elements, *A,* with three additional elements attached that copy its outputs. In its current state, subset *A* forms a main complex, and generates 3 bits of integrated information. However, the entire system does not form a complex: *φ*(*x*_1_) = 0 since the interactions outside of *A* are redundant. Elements {*n*_4_, *n*_5_, *n*_6_} are analogous to photodiodes in a digital camera, taking a snapshot of *A*'s state. The snapshot generates no integrated information over and above the original. Clearly an interaction occurs between elements *n*_3_ and *n*_6_, but from the perspective of the entire system it is redundant. Restricting attention to subset *B*, the couple, we see that integrated information generated by *B* is 1 bit.

#### Complexes must be analyzed at the level of elementary components and operations

**Figure 7:**
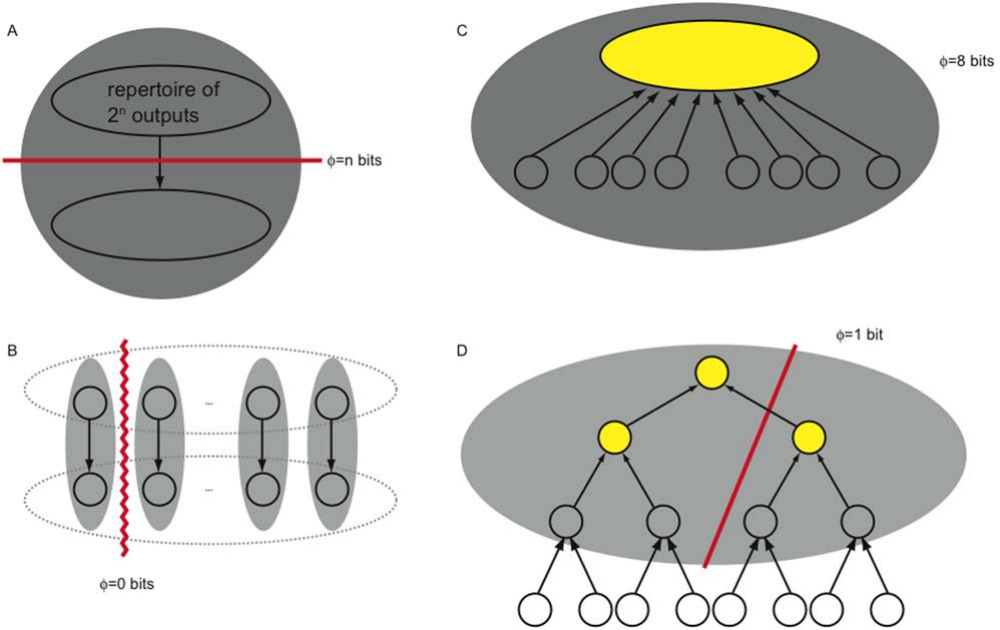
Analyzing systems in terms of elementary components. (A) and (C) show systems that on the surface appear to generate a large amount of integrated information. The units in (A) have a repertoire of 2*^n^* outputs, with the bottom unit copying the top. Integrated information is *n* bits. Analyzing the internal structure of the system in (B) we find *n* disjoint couples, each integrating 1 bit of information; the entire system however is not integrated. (C) shows a system of binary units. The top unit receives inputs from 8 other units and performs an *AND*-gate like operation, firing if and only if all 8 inputs are spikes. Increasing the number of inputs appears to easily increase *φ* without limit. (D) examines a possible implementation of the internal architecture of the top unit using binary *AND*-gates. The architecture has a bottleneck, shown in red, so that *φ* = 1 bit no matter the number of input units.

Finding the integrated information generated by a system requires analyzing it into complexes from the ground up in terms of elementary components and elementary mechanisms or operations. Figure 7 shows two examples of systems that appear to generate a large amount of integrated information, but on closer analysis dissolve into many weakly interacting components with low *φ*.

Consider the system in Figure 7A. If we ignore internal structure, we might assume that the system is made up of two components, each with a repertoire of 2*^n^* outputs. If the lower component copies the output of the upper in the previous time step then this two unit system generates *n* bits of integrated information – it would seem to be trivial to implement systems with arbitrarily large values of *φ*. However, we need to consider how such components could be built. Figure 7B depicts a simple construction: each component contains *n* binary elements, and the connection between the components decomposes into couplings between pairs of elements. Analyzing the system at this more detailed level uncovers a collection of disjoint couples each of which forms an independent complex and generates 1 bit of integrated information. Since the system as a whole is disconnected, *φ* = 0 bits. The dotted elliptic components have been artificially imposed on the system and do not reflect the underlying causal interactions, resulting in an incorrect value of *φ* in the higher-level analysis. Note that, if we attempt to address this problem by adding horizontal connections between elements, so that the components are integrated within, we introduce a second problem: the horizontal couplings shrink the *a posteriori* repertoires of the components, reducing effective information between them. We discuss a related example, and similar considerations for continuous systems, in Text S1, sections 10 and 11.

Figure 7C presents a similar situation. The system contains nine binary components, with a single component receiving inputs from the other eight; the component fires if all eight inputs are active in the previous time step. The minimum information partition is the total partition *P* = {*X*} and *φ*(*x*_1_) = 8 bits when the top component is firing, since it uniquely specifies the prior state of the other eight components. Increasing the number of inputs feeding into the top component while maintaining the same rule – fire if and only if all inputs are active – seems to provide a method for constructing systems with high *φ* using binary components. The difficulty once again lies in physically implementing a component that processes *n* inputs at a single point in space and at a single instant in time for large *n*. Figure 7D shows a possible internal architecture of the component, constructed using a hierarchy of logical *AND*-gates. When analyzed at this level, it is apparent that the system generates 1 bit of integrated information regardless of the number of inputs that feed into the top component, since the bipartition framed by the red cut forms a bottleneck.

The examples in this paper assume that the elements are abstract indivisible objects and that the rules are simple (logic gates, threshold functions and variations thereof). In future work we will investigate the internal structure of elements and determine the conditions under which they can be considered to be indivisible.

#### Extrinsic inputs can contribute to integrated information within a complex

The *a posteriori* repertoire of a complex X is specified using only information that is intrinsic to the complex; extrinsic inputs from the environment *E* = *W* \ *X* are averaged over and treated as extrinsic noise. At first glance it appears that environmental inputs cannot meaningfully contribute to the integrated information generated by *X*, however this is *not* the case.

**Figure 8:**
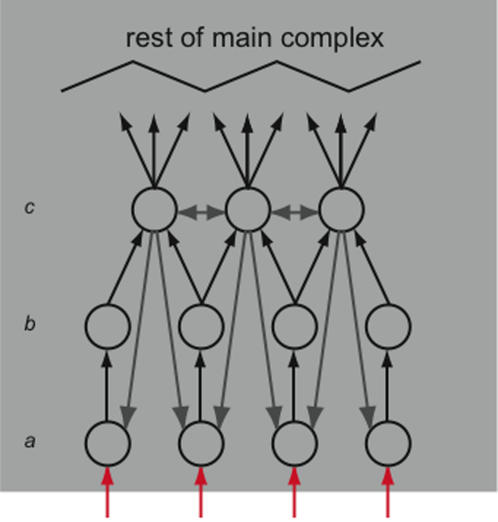
Integrated information and extrinsic inputs. The gray box represents a main complex. Red arrows are input from the environment. Black arrows depict strong feedforward connections; gray arrows are weaker modulatory connections. The black zig-zag represents the bulk of the main complex. The current state of row *R^a^* is determined by extrinsic inputs, which are treated as extrinsic noise. However the current state of row *R^b^* together with the feedforward architecture of the system together specify the prior state of *R^a^*, so that the system is able to distinguish extrinsic inputs *once they have caused an interaction between elements within the main complex*. Similarly row *R^c^* specifies higher-order invariants in the prior state of row *R^b^*.

Consider the cartoon example shown in Figure 8. The gray box is a main complex, with environmental inputs (red arrows) entering at the bottom. The bulk of the main complex (the black zig-zag) is not shown. The portion depicted can be considered, for example, as an idealization of the visual center of the mammalian cortex. It is dominated by strong feedforward connections driving the elements, with weak feedback and lateral connections. The system enters state *x*_1_. To what extent does the *a posteriori* repertoire of the system reflect environmental inputs?

We answer the question by considering the contribution of the current state of three rows of interest, labeled *R^a^* through *R^c^*, to the *a posteriori* repertoire. State 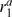 is entirely determined by the feedforward connections from the environment. External inputs are treated as noise, so the state 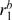 does nothing to reduce uncertainty regarding the *a priori* repertoire of states on *X*. Now consider the state 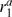. As shown, *R^b^* simply copies *R^a^*, so 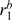 exactly specifies the prior state of *R^a^*. If *R^a^* is also copying its inputs, then the environmental inputs contribute to the information integrated by the system, albeit one temporal and spatial step removed. Row *R^a^* indirectly contributes to the total integrated information through the effect it has on *R^b^*. Finally *R^c^* specifies higher-order invariants in the *a priori states* of *R^b^* by combining them in some non-trivial manner. The *a posteriori* repertoire *p*(*X*_0_ → *x*_1_) reflects environmental inputs from time *t* = −1, and extracts higher-order invariants from environmental inputs at time *t* = −2. Therefore, a complex can reflect environmental inputs once they have resulted in causal interactions among its own elements.

## Results

We present examples and discussion, investigating the relationship between integrated information and network dynamics, causal architecture, and connectivity. Unless otherwise specified, for computational reasons we measure integrated information by considering all *bipartitions* rather than all partitions of a system. It is reasonable to do so since, as shown in the Text S1, section 6, restricting to bipartitions provides a lower bound on the expected value of integrated information. Further, in analyzing the basic examples below we are primarily interested in how causal interactions change as a function of network properties, rather than in the precise nature of the optimal modularization.

### Integrated Information Is a Function of Network Dynamics, Under a Fixed Causal Architecture

**Figure 9:**
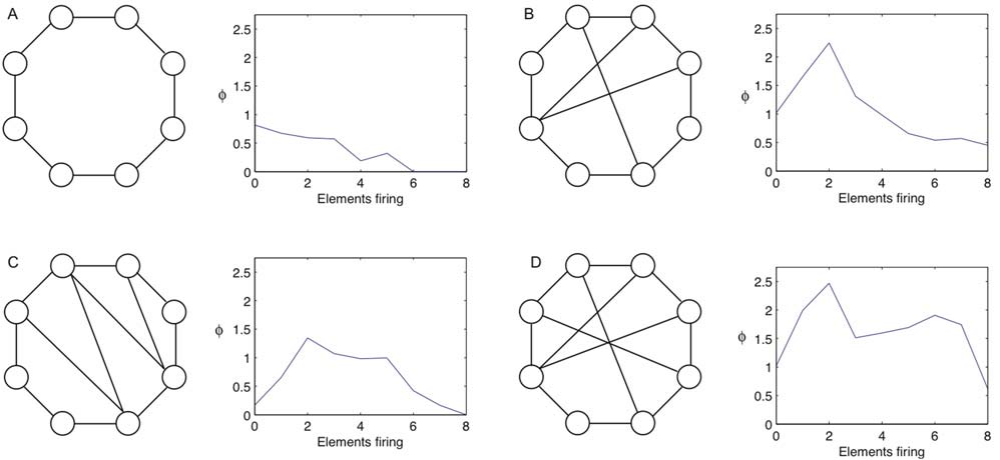
Integrated information peaks in balanced states. (A–D) show four discrete systems; lines represent bi-directional connections. Elements fire if they receive two or more spikes. The graph shows integrated information as a function of the number of elements firing. Integrated information is computed by averaging over all states with a particular number of elements firing. Integrated information is low for hyperactive and inactive states when too many or too few elements are firing, and high for balanced states lying between the two extremes. Note that in (A) the value of *φ* for 7 elements firing is undefined, since no state with seven elements firing is possible given the causal architecture.

Figure 9 shows four discrete systems. Elements fire if they receive two or more spikes. We refer to the number of elements firing as the firing rate of the system. Graphed alongside each system is integrated information, computed across bipartitions, as a function of the firing rate. The graph shows average integrated information, averaged over all output states (that can arise from the dynamics of the system) with the given firing rate.

#### *φ* is low in inactive and hyperactive states, and high when firing patterns are balanced

It can be seen from the 4 panels in the figure that inactive states – with no elements firing – are typically associated with low values of integrated information. This is because AND­-gates generate less information when silent than when spiking (compare Figure 1 and Figure 2B).

On the other hand, integrated information also decreases dramatically when the system is in a hyperactive state, with all elements firing. In Figure 9A and 9C no information is integrated because *too many elements are firing*. This obtains despite the fact that individual *AND*-gates generate more information when they are spiking than when they are silent. The reason integrated information is low when all elements are firing is that the system is “over-determined” and the whole adds nothing to the information generated by the parts. The highest values of *φ* occur for states with intermediate firing rates, which we refer to as balanced states (as connection density increases in the networks, the peak shifts towards higher firing rates). High *φ* means that many alternatives are ruled out by the entire system, and the parts are comparatively ineffective at specifying causes. Balanced states generate high *φ* because the output state of the system is highly flexible in its local causes and extremely rigid globally. The delicate trade-off between local flexibility and global rigidity justifies the term balanced.

Note that the systems in the figure generate (minimal amounts of) integrated information even when they remain inactive for long periods. Remaining in the inactive state requires that the elements rule out alternatives from the *a priori* repertoire. In contrast, when hyperactive the systems in Figure 9A and 9C generate *no* integrated information. Thus, depending on the architecture of the system in question, integrated information may potentially be generated even when the system is in a fixed state and appears to be doing nothing.

To a first approximation, cortical neurons can be considered to be roughly analogous to the *AND*-gates in the figure: they fire if a sufficiently large number of inputs are active within a given window of time. Thus, these observations may have some relevance concerning the relationship between neuronal firing rates in the thalamocortical system and consciousness. Consciousness is typically reduced when neuronal activity in the human brain is severely depressed, as under deep anesthesia or in certain comatose states. Though changes in brain function that occur in these conditions are not limited to a reduction in neuronal firing rates, the analysis of the figure indicates that integrated information would certainly suffer.

Consciousness also lapses when neuronal activity is excessive and hypersynchronous, as is the case in generalized seizures. The simple models shown in the figure suggest that the brain's capacity to generate integrated information would collapse also when the great majority of neurons were firing simultaneously.

Under normal conditions, cortical neurons *in vivo* operate in a finely balanced regime, hovering near their spiking threshold, with excitatory and inhibitory inputs approximately canceling each other out [12]–[15]. Maintaining a balanced level of firing must be exceedingly important for brain function, as the largest fraction of the brain's energy budget is devoted to sustaining spontaneous activity [16],[17]. The analysis of Figure 9 suggests that a fine balance between excitation and inhibition is required for a system to have high *φ*. Perhaps one reason why spontaneous activity is so important is that, by ensuring the availability of a large repertoire of causal states, it represents a necessary condition for a high level of consciousness.

#### High values of *φ* cannot be sustained under bistable dynamics

**Figure 10:**
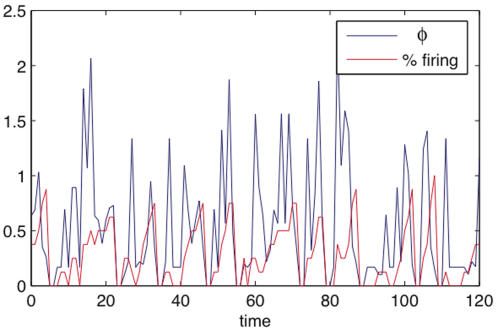
Bistable dynamics. The system has connectivity as in Figure 9C, with altered element behavior. If an element receives less than two spikes it fires with probability .15. If it receives 2 or more spikes it fires with certainty, unless more than half the elements fired in the two times step prior, in which case all elements are silent. The graph plots *φ* and the percentage of elements firing, as the system is run for 120 time steps. The system implements a bistable dynamics, and is unable to sustain high values of *φ*.

Figure 10 investigates a modified version of the network in Figure 9C. The connectivity is unchanged, but the rules are altered to implement a bistable dynamics. The hyperactive and inactive states are made unstable, resulting in the system oscillating between the two extremes; see figure legend for details. The red curve in Figure 10 shows the percentage of elements firing. The network tends to remain at low firing rates for a while, until a threshold of three or more elements firing is reached. Elements are then rapidly recruited into the firing pattern until the system approaches a hyperactive state and activity is shut down. The blue curve shows *φ*, which reflects the bistable dynamics. *φ* peaks when a few of the elements are firing. However, it collapses to nearly zero when the system is hyperactive, or when the elements are shut down. The jagged form of the curve is a consequence of the network having only eight elements, resulting in abrupt dynamics.

Despite its simplistic implementation, the bistable behavior of the simulated network bears some resemblance to that of thalamocortical circuits during slow wave sleep [18]. Throughout slow wave sleep, all cortical neurons alternate between a depolarized UP state during which spikes are generated, and a hyperpolarized DOWN states during which there is complete synaptic inactivity. The alternation between UP and DOWN states, which happens on average once a second, reflects the underlying bistability of cortical circuits, as suggested both by experimental perturbations [19] and by detailed models [20]. During wakefulness, by contrast, thalamocortical circuits return to a state of balanced depolarization and tonic firing. Based on the present analysis, it would appear that, when thalamocortical circuits become bistable, they cannot sustain high levels of integrated information, which could in principle account for the fading of consciousness during early sleep [21].

### Integrated Information Is a Function of Causal Architecture Under a Fixed Network Dynamics

#### *φ* can vary in systems with identical surface dynamics (cycling through the same states in the same sequence) depending on the presence/absence of causal interactions

**Figure 11:**
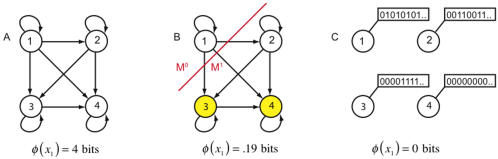
System cycling (via binary counting) through 16 firing patterns. The system cycles through the firing patterns 0000, 0001, 0010, …, 1101, 1110, 1111; counting in binary from 0 to 15 and repeating. (A) and (B) show the system, implemented with interacting memoryless elements, in two different states. (C) shows a system with identical dynamics, implemented using four elements independently replaying a list of instructions. Since there are no causal interactions, the replay generates no integrated information, in contrast to the memoryless system.

Figure 11 depicts a system of four elements. Suppose the system cycles through all 16 possible firing patterns as follows: 0000, 0001,…,1111, 0000, counting in binary from 0 to 15 and then repeating. Consider two ways in which the dynamics could be implemented. The first uses memoryless elements, with connectivity as shown in the figure. For example element *n*_2_ has afferents from itself and *n*_1_, and fires if it receives input pattern (*n*_1_, *n*_2_) = (0,1) or (1,0), and is silent otherwise. Alternatively, the same dynamics can be achieved with no interactions between the elements, as in Figure 11C. Element *n*_1_ alternates between firing 0 and 1. Element *n*_2_ alternates firing 00 and 11, and so forth. Element *n*_1_ has a memory of one time step, and element *n*_4_, which alternates firing eight consecutive zeros and ones, has a memory of eight time steps. The two implementations (with correct initial conditions) produce identical dynamics, and cannot be distinguished by observing the systems. Nevertheless, in the first case *φ* = 4 (across the total partition) or *φ* = .19 bits, depending on the current state, and in the second case it is zero for all states.

More generally, suppose we have two systems exhibiting complex, yet identical, behavior. The first contains many interacting elements, the second is a “replay”: each element is given a list of outputs that it runs through, reproducing the original behavior. Passively observing the two systems and computing correlations, mutual information or related quantities does not distinguish between causal interactions and mere replay. In contrast, perturbing the systems and measuring integrated information does discriminate between the two situations. No integrated information is generated by the replay, and (potentially) high integrated information is generated by the causally interacting system.

#### *φ* can vary in systems with similar surface dynamics (cycling through the same states in a different sequence) depending on the complexity of the causal interactions

Behaviors that appear qualitatively similar may require very different causal architectures. Suppose we scramble the sequence of firing patterns to the following:
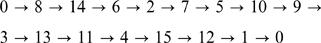
where 0 corresponds to firing pattern 0000, 6 to 0110, and so forth. The dynamics are qualitatively the same in that the system cycles through all 16 possible firing patterns as before; all that has changed is the sequence. Nevertheless, computing *φ* for this network we find that it is 4 bits for all states, with the total partition as the *MIP*. The original counting sequence was implemented in a memoryless system using the connectivity shown in Figure 11. The Boolean functions implemented by the elements become progressively more complicated going from *n*_1_ to *n*_4_ as the elements require more information to keep track of their position in the sequence. Nevertheless the system admits a simple description – binary counting – which the architecture reflects. The scrambled sequence does not admit a simple description and implementing it with memoryless elements requires denser wiring and more complicated Boolean functions (see Text S1, section 12).

From a dynamical systems point of view the counting sequence and the scrambled version are difficult to distinguish. The phase space is a four dimensional hypercube with 16 vertices corresponding to the 16 firing patterns. In both cases the system cycles through all the points in the hypercube. The difference, which *φ* is sensitive to, is in the details of the specific firing patterns and the causal interactions which lead to the system transitioning from one state to another. It is not immediately obvious from the dynamics how complicated the underlying interactions are, or indeed, whether the dynamics are a result of interactions at all. Integrated information captures the extent to which a systems dynamics are due to causal interactions within a single entity. This suggests that *φ* is related to the notion of an ε-machine [22] introduced in computational mechanics, which captures the minimal causal architecture that can generate a given pattern. Similarly, the algorithmic complexity [23] of the processing within a system, where the algorithmic complexity of an object (in our case the interactions within the system) is a measure of the computational resources required to describe it, should be related to the integrated information generated by the system.

### Integrated Information Is a Function of Causal Architecture

#### High *φ* requires functionally integrated and functionally specialized networks

**Figure 12:**
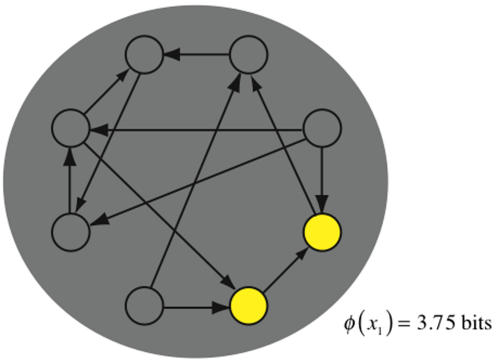
Optimized network of *AND*-gates. The network is optimized to generate high integrated information in a single state, that shown. Each element implements an *AND*-gate.

We have seen that, for a fixed causal architecture, the dynamics of a system determines the quantity of integrated information generated. This section shifts the emphasis and considers how integrated information depends on the underlying causal architecture. A system optimized to generate high *φ* using simple rules (*AND*-gates) is shown in Figure 12. The elements in the network are limited to receiving exactly two inputs (or zero in the case of the two “sources”). The system generates *φ* = 3.75 bits for the firing pattern shown. The network is densely and heterogeneously connected. Although every element applies the same rule, they are functionally specialized by virtue of their varying connectivity: the elements play distinct functional roles in the network, receiving unique sets of inputs, and thus specifying the *a posteriori* repertoire in different ways. The system is functionally integrated since it does not decompose into disconnected pieces or into weakly connected modules: the architecture tightly binds the elements, in spite of the sparse connectivity. Further, the optimized architecture is recurrent: there are multiple feedback loops embedded in the system.

It was not possible to scale the architecture up to more than a few elements because of the computational burden entailed in optimizing *φ* for large systems, although there is evidence to suggest that architectures balancing functional specialization with functional integration produce complex dynamics [24], and so may be able to generate high *φ*. In the remainder of the section we investigate the general consequences of imposing structural restrictions on the class of networks under consideration (imposing strongly modular or homogeneous architectures for example) and describe the resulting information-theoretic bottlenecks, regardless of network size.

#### *φ* is low for strongly modular systems

**Figure 13:**
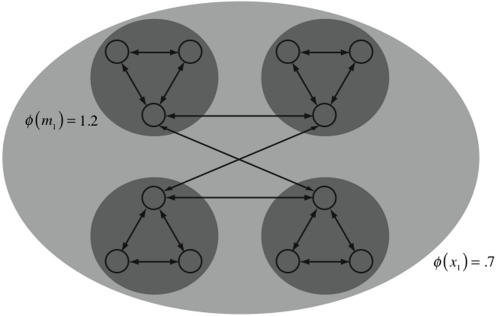
Integrated information in a strongly modular network. The system is composed of three four-element modules. The elements fire if they receive two or more spikes. The entire system forms a complex (light gray) with *φ*(*x*_1_) = .7 bits; however, the architecture is strongly modular so that the main complexes (dark gray) are the modules, each generating *φ* = 1.2 bits of integrated information across the total partition, more than the system as a whole.

Figure 3 presented an example of a perfectly modular system: the two couples were disconnected. Each couple formed a complex and no integrated information was generated by the entire system. More generally, we can consider strongly modular systems, in which there are weak or sparse connections between highly integrated modules. Figure 13 shows a strongly modular system of four modules, with all elements silent. Each module is reciprocally connected to two of the others. Integrated information is low; in particular, each of the modules generates *φ*(*m*_1_) = 1.2 bits of integrated information. In contrast to the couples, the whole system does form a complex, but *φ* is .7 bits, even lower than for the modules. Simply connecting a collection of large integrated modules together does not ensure the resulting system will have high *φ*. It is necessary that the modules be properly integrated with one another. In strongly modular systems the weak or limited interactions between modules forms a bottleneck, keeping *φ* low as in the figure.

#### *φ* is at most one bit for homogeneous networks with binary elements

Strongly modular systems suffer from the defect that they are insufficiently integrated. At the opposite end of the spectrum are homogeneous systems, which lack specialization. A homogeneous network has all-to-all connectivity, including self-connections. There are no limitations on the computational power of the elements, and connections efferent to different elements can have different weights, but we require that all the connections efferent to a given element be identical and that all elements implement the same computation. Under these conditions, the maximum expected integrated information generated by the system is 1 bit (see Text S1, section 6).

**Figure 14:**
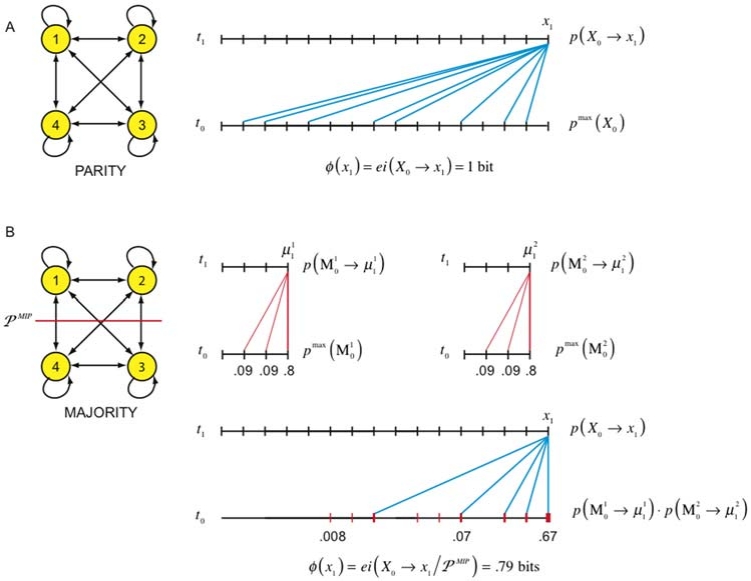
Integrated information in homogeneous systems. The systems have all-to-all connectivity, including self-connections. (A) shows a parity system: each element fires if it receives an odd number of spikes and is silent otherwise. The *MIP* is the total partition and integrated information is 1 bit. (B) shows a majority-rule system where elements fire if they receive three or more spikes. The *MIP* is the bipartition shown. The *a posteriori* repertoire specified by each part contains three perturbations, with weights .09, .09, and .8 respectively. The combined *a posteriori* repertoire contains 9 perturbations of varying weights, as shown. The *a posteriori* repertoire of the system contains 5 equally weighted perturbations. Integrated information is .79 bits.

Figure 14A is an example of a homogeneous system, the parity system. Elements fire if they receive an odd number of spikes and are silent otherwise, so that perturbing any element changes the output of every element in the next time step. It follows that no part is independently able to rule out any alternatives: the *a posteriori* repertoires of the parts is the maximum entropy distribution, and 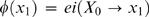. The *a posteriori* repertoire of the system specifies whether the prior state was even or odd, generating 1 bit of information, thus for the parity system, *φ* = 1 bit. Increasing the number of elements in the parity system makes no difference to the amount of integrated information it generates.

Figure 14B shows a second homogeneous system, operating according to a majority rule: elements fire if more than half fired in the previous instant. The minimum information partition is given by a vertical or horizontal bipartition, shown in the figure. In contrast to the parity system the parts are able to partially reduce uncertainty independently, so *φ* is even lower, .79 bits.

#### *φ* is at most proportional to *n* for an *n*×*n* lattice of binary elements

**Figure 15:**
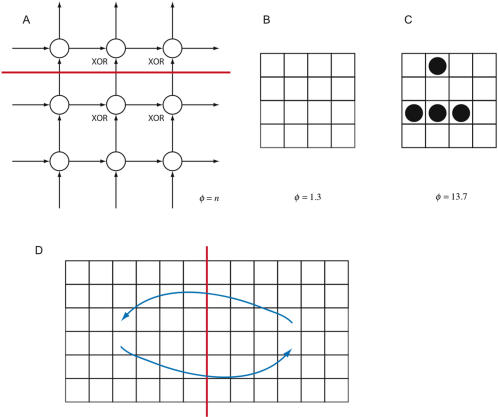
Integrated information for lattice architectures. (A) is an *n*×*n XOR*-lattice. The minimum information partition is given by a vertical or horizontal midpartition. Integrated information is *n* bits; and so can be increased without limit by scaling up the (highly inefficient) architecture. (B) and (C) show integrated information for a Game of Life grid in two different configurations. Cells in the grid are either ON or OFF. Each cell has 8 neighbors, the grid is assumed to wrap around to form a torus. A cell that is OFF switches to ON in the next time step if exactly 3 of its neighbors are ON. An ON cell remains ON if two or three neighbors are ON; otherwise it switches to OFF. (D) shows long-range connections short-circuiting the perimeter bottleneck intrinsic to lattice architectures.

Figure 15 shows two causal architectures that can be implemented on two-dimensional lattices. Figure 15A shows a grid consisting of *XOR*-gates. The *XOR*-grid has minimum information bipartition given by a horizontal or vertical mid-partition, so for a system with *n* ^2^ elements we find *φ* = *n* bits, regardless of the state the system enters. The main complex is the entire grid.

Figures 15B and 15C show integrated information generated by a Game of Life grid [25] in two different configurations. As with the *XOR*-grid, *φ* on *n*×*n* Game of Life grid is approximately (depending on the configuration) proportional to *n*. It is known that a universal Turing machine can be built on an infinite Game of Life grid. Thus we should not be surprised that *φ* can increase without bound as a function of grid size: there is no limit to the computational power that can be embedded in lattice architectures. In particular, for the Game of Life, this suggests that certain configurations act in concert so the system admits a higher-order description. Forthcoming work will present a framework for analyzing systems at different spatiotemporal scales and apply it to uncover higher-order structure in the Game of Life; we will further show that the *XOR*-grid possesses *no* higher-order structure.

It is possible, but inefficient, to build systems with high *φ* using grid architectures. As a point of comparison, the 8-element *AND*-gate network in Figure 12 generates *φ* = 3.75 bits, considerably more than the maximum attained (2.3 bits) by a 3×3 grid of *AND*-gates. The inefficiency increases with the size of the grid; for example an *XOR*-grid of a million elements is needed to generate 1000 bits of integrated information. *φ* of a grid is limited by the interactions occurring along the perimeters of the parts, so that the expected value of *φ* for an *n*×*n* grid is proportional to *n* (see Text S1, section 6). More generally, in a three-dimensional lattice interactions occur along the surfaces of the parts, so *φ* will be proportional to their surface area, a phenomenon similar to the holographic principle [26].

Introducing longer range connections, as in Figure 15D, short-circuits the perimeter bottleneck by exposing the interiors of the minimal parts to interactions with one another, and may (depending on the elements) increase *φ*. These additional connections are biologically realistic: neurons in the cortex have dense local connectivity and sparser links to distal regions, and can be idealized as forming a grid with long-range connections.

#### Strict feedforward architectures are inefficient for generating integrated information

**Figure 16:**
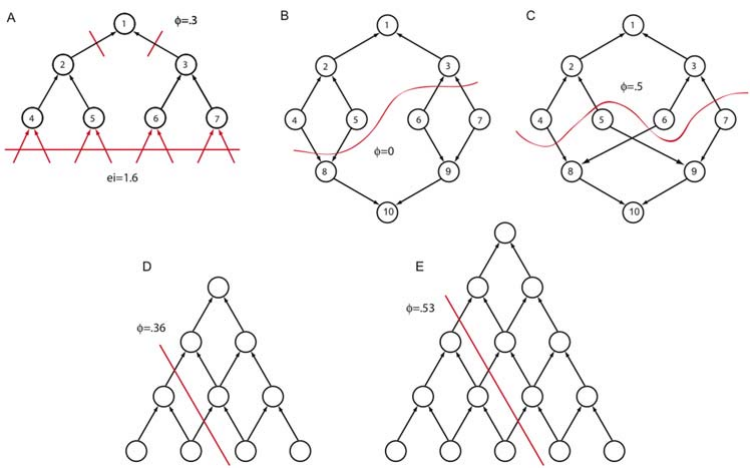
Integrated information in feedforward networks. (A) shows a tree-like hierarchical feedforward network. Effective information from the sensory sheet below the grid (not shown) is high, 1.6 bits, but the ability of the network to integrate information is limited by the bottleneck at the grandmother cell *n*_1_. (B) and (C) show the network with an additional grandmother cell (the network is flattened out, so the second grandmother appears at the bottom). A redundant grandmother results in zero integrated information, whereas if the grandmother is *not* redundant *φ* increase to .5 bits. (D) and (E) depict a grid-like feedforward architecture that does not suffer from the bottleneck of (A). Integrated information increases with network size.

Strictly feedforward networks, Figure 16, are commonly used in pattern recognition tasks. A sensory sheet below the network feeds inputs into the system. Effective information between the system in Figure 16A and the sensory sheet is 1.6 bits or 8 bits, if all or none of the elements in the bottom layer is firing respectively. A more realistic network could be designed using more elements with greater computational power, resulting in higher values of effective information generated across the sensory sheet. However, the sensory sheet does not determine the integrated information generated by the system; instead we need to find the minimum information partition. In Figure 16A the *MIP* is given the cut separating the grandmother element *n*_1_ from its inputs. *φ* = .3 bits when no elements are firing and *φ* = 1 bit when all elements are firing. Increasing the size of the network makes no significant difference so long as the bottleneck introduced by the tree-like hierarchical architecture is in place.

Figure 16B and 16C consider the effect of adding additional elements and connections to create a second grandmother cell. If the second grandmother is redundant, extracting identical invariants to the first, Figure 16B, then *φ* = 0 bits. If the second grandmother extracts different invariants, as in Figure 16C, then integrated information increases from *φ* = .3 to *φ* = .5.

Finally in Figure 16D and 16E we consider grid-like feedforward architectures that have additional connections breaking the tree structure of the previous examples. These networks do not suffer from a bottleneck at the grandmother cell, and *φ* increases with network size. However the networks are diagonal sections of a grid, and so are similarly inefficient at generating integrated information. Adding feedback and lateral connections to a feedforward network can potentially increase the integrated information generated by a system by increasing the number of non-redundant causal interactions.

### Integrated Information for Probabilistic Systems (Hopfield Networks)

Small synchronously updated Hopfield networks [27],[28] provide a class of examples that are computationally tractable and have interesting dynamics. Hopfield networks are probabilistic systems constructed so that for any initial condition the network tends to one of a few stable firing patterns called attractors. The integrated information generated by a firing pattern depends, in an interesting way, on the relationship between the firing pattern and the attractors embedded in the network.

A Hopfield network consists of *N* elements with all-to-all connectivity. The probability of the *i^th^* element firing at time *t* is given by
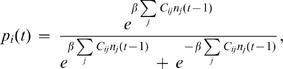
where *n_j_*(*t*−1) is 0 or 1 according to whether the *j^th^* element fired at time *t*−1; and *β* = 1/*T* The temperature *T* is a measure of the amount of indeterminacy in the system: *higher temperatures correspond to more noise*. The connection matrix *C_ij_* is constructed so that the network contains certain attractors. For each attractor stored deliberately there will be additional “spurious” attractors: for example a network designed to store the firing pattern {0…01…1} will also contain its mirror image {1…10…0}. This is a quirk of the Hopfield network design. The construction is as follows. Suppose we wish to store attractor states *ξ* ^1^,…*ξ^P^*. Set 
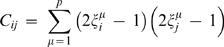
 With this connection matrix and the probabilistic firing rule above the network will typically – depending largely on the temperature – settle into one of the attractor states (including the spurious states) given any initial condition. The construction crucially depends on the near orthogonality of the attractors considered as vectors in the *N* dimensional space determined by the network. Choosing *N* to be large – hundreds of elements – and picking the attractors randomly, most easily arranges this near orthogonality. Since Hopfield networks possess all-to-all connectivity and identical elements they are similar to homogeneous systems. The crucial difference is that the weights on the arrows afferent to each element vary.

**Figure 17:**
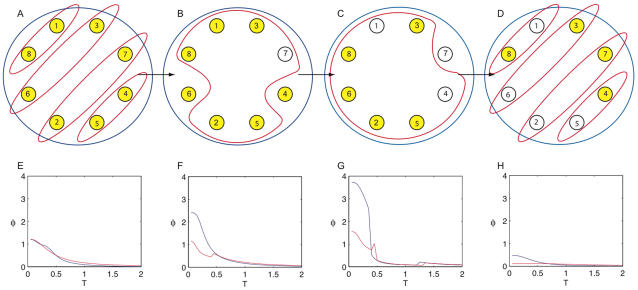
Integrated information in a Hopfield network. (A-D) show a sample run of a Hopfield network with 8 elements and all-to-all connectivity. The network has embedded attractors 11110000, 11001100, 10101010 and their mirror images. A sample run is depicted at *T* = .45 and initial state 11111111. (E-G) show integrated information as a function of temperature (computed using bipartitions) for the corresponding states; the colored enclosures are matched with the graphs. The system forms a complex for low temperatures (blue), but breaks down for higher temperatures (red), so that subcomplexes arise.

Figure 17 depicts a Hopfield network consisting of 8 elements with 6 embedded attractors. Since we work with a small network randomly chosen attractors will not be orthogonal; instead we carefully choose the attractors so the patterns do no interfere with one another. The attractors are 00001111, 00110011, 01010101, and their mirror images. A sample run is shown at temperature *T* = .45 and initial state 11111111. The network quickly relaxes into an attractor. The graphs show integrated information as a function of temperature, which ranges between .05 and 2. We analyze integrated information generated by the system in detail for different states to better understand how integrated information and the repertoire reflect the dynamics.

The specific choice of attractors has an interesting consequence. Notice that the pairs of elements *n*_1_ and *n*_8_ have opposite outputs in all 6 attractors. Similarly for the pairs {*n*_2,_ *n*_7_}, {*n*_3_, *n*_6_}, and {*n*_4_, *n*_5_}. It turns out that the connection matrix for the Hopfield network (embedding the 6 attractors above) has stronger connections within each couple than between them. The couples are the dominant small-scale feature of the network, a structural feature that will be reflected in the integrated information generated by the system.

#### *φ* varies with temperature in a Hopfield network

First, the graphs in Figure 17 show that *φ* decreases as temperature goes up. The increase in noise reduces the systems' ability to specify their *a posteriori* repertoires. Second, the trend across panels is that the size of the main complex decreases with increased temperature. In Figure 17B the main complex shrinks from the entire system to a 6 element subset, and in Figure 17A and 17D it decomposes into a collection of couples. Intuitively, at higher temperatures the system is less able to cohere: it becomes less and less reasonable to treat it as a single entity. This is reflected in the decreased values of *φ* and more fundamentally in the reduction in size of the main complex.

#### *φ* is low for attractor states and neutral states in a Hopfield network

Of all the Hopfield network's 256 possible firing patterns, integrated information is lowest when the system is in the states depicted in Figure 17A and 17D. The 6 attractors are the firing patterns with the lowest *φ*. If we take an attractor state and alter it minimally, respecting the couple structure, we find we have to change two elements at once. There are 8 ways of doing this, producing firing patterns such as 00011110, 00101011 and so forth. These form the category of firing patterns providing the second lowest values of *φ*, along with a second group that we now describe. The second group contains 16 firing patterns, including 11111111; we term states in this category neutral states. The defining characteristic of neutral states is that both elements in each couple have *the same* output.

Why do attractors and their neighbors generate low integrated information? When the system is in an attractor all of its elements are acting in concert to reinforce the current state. The parts are able to independently rule out most *a priori* states, and the interactions between the parts provide little extra information. Neutral states are far removed from the attractor states, and the causal architecture of the system is strongly biased against these states occurring, particularly in systems with low temperature. They are highly unstable. Nevertheless, unlike the tense states described below, *φ* is low. Neutral states are locally incompatible with the architecture of the system, since the elements in each couple have the same state, an outcome in opposition to the network's connectivity. The elements are working against each other locally (at the level of couples), and so the system does not cohere as a single global entity.

#### *φ* is high for tense states in a Hopfield network

Tense states are the opposite of neutral states: they are locally compatible with the architecture of the system, but globally incompatible. The global tension is grounded and amplified by the local compatibility. The tense states are 01101001 and 10010110. These resemble the attractors in that each couple is in its natural, internally opposed, state. However, of all states that respect the internal structure of the couples, they are the most different from the attractor states, differing in 4 elements. Thus, the state is compatible with the couples' causal architecture, but highly incompatible with the architecture of the entire system (the relations between couples). The state depicted in Figure 17C is a near-tense state, differing from a tense state in a single element, and thus generates a large amount of integrated information.

#### A functionally integrated and functionally specialized probabilistic network can sustain high *φ*

A massive Hopfield network with many embedded attractors may have high values of *φ* in certain states, but *φ* will rapidly decrease as the system relaxes into an attractor. The system is too tightly bound to sustain interesting dynamics: the elements act in concert to push the system into its attractors. To sustain high *φ* it is necessary to change the connectivity so that elements act antagonistically.

**Figure 18:**
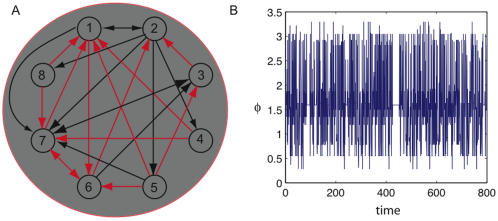
A functionally integrated and functionally specialized probabilistic network can sustain high *φ*. (A) shows a functionally integrated and functionally specialized network; black arrows represent connections of weight ≥.5 and red arrows connections with weight ≤−.5. Weaker connections are not shown to reduce clutter; see Text S1, section 13. The elements operate according to the rules of a Hopfield network with *T* = .35. The network is initialized with a random firing patter and allowed to run for 800 time steps. (B) shows *φ* for each firing pattern that occurs during the run.

The network in Figure 18 has Hopfield-like elements. The system differs from Hopfield networks in that it is *not* constructed to store attractor patterns. It was optimized using a genetic algorithm, searching through networks with approximately 50% of full connectivity to find those most capable of sustaining high values of *φ*. The algorithm compares different networks by initializing them with a random firing pattern, running for 125 time steps and calculating *φ* for 10 odd firing patterns that occur. The temperature is fixed at *T* = .35 throughout. Over an 800 time step simulation we find that 109 of the 256 possible firing patterns arise. Of these, the 14 most common occupy slightly more than half of the running time. The system does not possess any attractors, but the dynamics are dominated by a small number of characteristic firing patterns. For each time step we compute *φ*, which varies with the state the system enters. The graph shows *φ* as a function of time; values range between .25 and 2.9 bits. Values greater than 1.1 bits occur 70% of the time, and *φ* of over .7 bits is generated 90% of the time. Contrast to a Hopfield network, which would remain close to an attractor over the entire run, with *φ* around .3 bits.

From this example it appears that the optimization produces a similar architecture to that shown in Figure 12. The introduction of noise produces a looser system that does not become trapped in fixed-points; with the trade-off being a reduced ability to specify sharp *a posteriori* repertoires. Again, although the elements all implement the same rule, the heterogeneous connectivity results in functional specialization. In addition the network is densely connected, leading to functional integration. The asymmetric, antagonistic connectivity prevents the system from relaxing into an attractor state and produces sustained “tense” dynamics in the network, and the system is thus able to consistently generate high values of integrated information. This suggests that metastable systems [29] – characterized by antagonism between the connectivity within and across neuronal groups, and capable of switching rapidly between states – may form an interesting class of systems with high *φ*.

## Discussion

In the paper we have extended the notions of effective information and integrated information to discrete non-stationary systems. Integrated information, *φ*, measures the information generated by a system above and beyond what can be accounted for by its parts acting independently. The subsets of a system capable of generating integrated information form complexes, which constitute individual entities and are the fundamental units into which a system decomposes. Finding the integrated information generated by a physical system requires analyzing it from the ground up, without preconception regarding the nature of its elementary units.

In the applications we analyzed a variety of systems to uncover how *φ* reflects network dynamics and architecture. A few broad lessons can be extracted. First, the integrated information generated by a system depends on the current state of the system. In general, integrated information is higher when there is a balance between the number of active and inactive elements of a system. By contrast, when a system is completely inactive or hyperactive, *φ* values are low. Second, integrated information can differ substantially for systems with identical or similar surface dynamics, because the latter does not necessarily reflect the causal architecture of a system. For instance, a system composed of causally interacting elements can generate large amounts of integrated information, while a mere copy or “replay” of its surface dynamics generates none. More generally, integrated information appears to be a function of the complexity of the interactions leading to the observed dynamics. Third, we observed that certain classes of network architectures have low *φ*. Modular and homogeneous systems are unable to generate high *φ* because the former lack integration whereas the latter lack information. Feedforward and lattice architectures are capable of generating high *φ*, but they are extremely inefficient. Everything else being equal, it appears that high values of integrated information can be obtained by architectures that conjoin functional specialization with functional integration. Finally, from the probabilistic (Hopfield-style network) examples we conclude that high *φ* can be produced by tension between local and global interactions. Conventional Hopfield networks relax into attractor states and so cannot sustain high *φ*. However, random Hopfield networks can be optimized to maintain higher values of *φ* over the course of their dynamics.

The notion of integrated information is motivated by the need for a measure that captures the two basic phenomenological properties of consciousness: *i)* each conscious experience generates a huge amount of information by virtue of being one of a vast repertoire of alternatives; and *ii)* each conscious state is integrated, meaning that it is experienced as a whole and does not decompose into independent parts. We have shown that the way *φ* behaves in simple simulated networks differing in causal architecture and dynamics fits available neurobiological evidence concerning the neural substrates of consciousness. For example, *φ* is low for simple network analogues of inactive (“comatose”) and hyperactive (“epileptic”) states, in line with the loss of consciousness when the brain enters such states. Conversely, high *φ requires balanced states* similar to those observed when the brain is spontaneously active during waking consciousness. We also saw that a simplified model of bistable dynamics, loosely resembling slow-wave sleep early in the night, when consciousness fades, is not able to sustain high values of integrated information. We provided evidence that, everything else being equal, causal architectures characterized by a coexistence of functional specialization and integration are best suited to generating high values of *φ*, whereas strongly modular systems fare much less well. Neurobiological evidence suggests that human consciousness is generated by the thalamocortical system [3], the paradigmatic example of a functionally specialized and functionally integrated network. The cerebellum, which is instead organized into strong local modules with little communication among them, does not seem to contribute to consciousness, though it is as rich in neurons and connections as the cerebral cortex. Finally, the analysis of Hopfield networks shows that tension between the local and global connectivity of a system results in high *φ*. This suggests that metastable systems, which arise when a collection of neuronal groups are loosely coupled, may be highly integrated. Intriguingly, some initial evidence obtained with multiunit recordings suggests that in awake, behaving animals populations of neurons may undergo a similar metastable dynamics [30],[31].

A few general observations about the present measure of integrated information are also in order. First, *φ* measures a process: integrated information is generated by a system transitioning from one state to the next – it does not make sense to ask about the information value of the state of a system *per se*. Second, *φ* is a causal measure: integrated information is generated only to the extent that the system transitions into a given state due to causal interactions among its elements. Thus, a system that enters a particular state due to extrinsic noise generates no integrated information, as in Figure 2D. The same is true for a system whose elements update their state without interacting, as in Figure 11. Importantly, causal interactions can only be made explicit by perturbing the system in all possible ways. Third, *φ* captures an intrinsic property of a system: integrated information is a function of the possible causal interactions within a system, independent of external observers. In this sense, integrated information is closer to other intrinsic properties of physical systems, such as charge or spin, than to observer-dependent properties that vary with the frame of reference, such as position or velocity. Specifically, integrated information is associated with and indeed identifies complexes – sets of elements that cannot be meaningfully decomposed into independent parts – independently of external observers. For example, elements forming two independent complexes may be lumped together into an externally defined “system” by an observer, as in Figure 7, but such arbitrary entities generate no integrated information – from an “intrinsic” perspective, they do not really exist. The intrinsic nature of integrated information, which only exists to the extent that it makes a difference from the perspective of the complex itself, is usefully contrasted with the traditional, observer-dependent definition of information, in which a set of signals are transmitted from a source to a receiver across a channel (or stored in a medium), and their “integration” is left to an external human interpreter.

Finally, we should mention some of the many limitations of the present work. Foremost among them is that our examples are restricted to small-scale models, so it is unclear to what extent the principles suggested by our partial explorations would scale with larger networks. The impossibility of measuring integrated information for larger systems is due to the combinatorial explosion in the partitions of a system as the number of elements is increased. An inevitable consequence is that computing *φ* for parts of the human brain, even if the connectivity and causal architecture of the neurons were known, is not a feasible undertaking, though heuristics, estimates, and relative comparisons remain possible. Applying the measure to biological system also introduces the practical issue of correctly identifying the causal architecture and *a priori* repertoire, a difficult empirical problem (for example, when dealing with neural networks, should all possible firing patterns be considered, including those differing by just a millisecond, or should they be lumped together?) From a theoretical perspective this problem should be addressed according to what may be called a principle of “causal ontology”: only those differences that make a difference within a system matter; differences between *a priori* perturbations that cannot be detected by the system can be considered as if not existing. There are a number of further issues that will be addressed in future work. A limitation of the present work is the exclusive focus on the amount of information integrated by a given network, with no consideration given to the kind of informational relationships among its elements. To address this we will move beyond quantifying integrated information as a single number and investigate the informational relationships between interacting parts by exposing the geometry of the causal interactions. Another shortcoming is that it focuses exclusively on memoryless systems, in which integrated information can only be generated over one time step. In a forthcoming paper we will coarse-grain discrete systems and develop techniques to find the natural spatiotemporal scale at which a system generates integrated information. This will allow us to deal with systems with memory, as well as to make a first step towards analyzing large-scale, hierarchically organized systems. Finally, the networks considered here were analyzed as isolated entities, without consideration for their environment (or rather by averaging over possible extrinsic inputs). In future work we will discuss how discrete systems interact with and incorporate information from the environment, as well as the relationship between integrated information and learning.

## Supporting Information

Text S1 Supplementary Information
(0.55 MB DOC)
