# Supplementary material for: Integrated Information in Discrete Dynamical Systems: Motivation and Theoretical Framework
Source: PLoS Comput Biol. 2008 Jun 13;4(6):e1000091. doi: 10.1371/journal.pcbi.1000091 (PMC2386970; doi:10.1371/journal.pcbi.1000091)
Supplement: Text S1 — Supplementary Information (0.55 MB DOC) [file pcbi.1000091.s001.doc]

# Supplementary Information

## *1.* Relative entropy and entropy

***Relative entropy.*** Given two probability distributions *p* and *q* defined on a set *Z* of $N$possible outcomes, the relative entropy [1] of *p* with respect to *q* is given by

$$H\left[p\text{ }\left\|\text{ }q\right.\right]:=\sum_{i=1}^{N} p_{i}\log_{2}\left(\frac{p_{i}}{q_{i}}\right).$$

Relative entropy is also known as Kullback-Leibler divergence. It can be shown that relative entropy is always non-negative, $0\leq H\left[p\left\|q\right.\right]$, and further that $H\left[p\left\|q\right.\right]=0$ if and only if $p=q.$ A special case arises in the context of Bayesian updating. Suppose $p(H)$ is the prior distribution for a set of hypotheses, and $p\left(H\left|D=d\right.\right)$ is the posterior distribution given some new data *d*. The relative entropy $H\left[p\left(H\left|D=d\right.\right)\text{ }\left\|\text{ }p(H)\right.\right]$ quantifies the number of bits of information about *H* that are gained by learning $D=d$.

We extend the interpretation to arbitrary distributions *p* and *q*, where *q* is a prior distribution and *p* is a posterior. We take a posterior distribution to be a distribution that is specified after causal interactions have taken place.

Relative entropy $H\left[p\left\|q\right.\right]$ measures the information generated by specifying *p*, relative to *q*. We emphasize that it is meaningless to measure the information generated by a repertoire *p* without a yardstick, given here by *q*.

***Entropy.*** It is useful to compare relative entropy to the more familiar notion of (information) entropy. Entropy is defined as

$$H\left(p\right):=-\sum_{i} p_{i}\log_{2}\left(p_{i}\right),$$

where we use round brackets to distinguish the two entropies. A related notion is the concept of self-information. Given a set of outcomes *Z* distributed according to *p*, the self-information given by outcome is $I\left(z_{i}\right):=-\log_{2}\left(p_{i}\right)$. Self-information is usually interpreted as a measure of the surprise associated with outcome $z_{i}$. If $p\left(z_{i}\right)=1$ then $I\left(z_{i}\right)=0$, there is no surprise; if $p\left(z_{i}\right)=0$ then $I\left(z_{i}\right)=\infty$, an impossible outcome is infinitely surprising. Comparing the formulae for entropy and self-information it is clear that entropy is the expected value of the self-information of *Z*.

We apply this as follows. Let $\delta_{i}$ be the probability distribution on *Z* assigning probability 1 to outcome  and probability 0 to all other outcomes. Observe that $I\left(z_{i}\right)=H\left[\delta_{i}\left\|p\right.\right]$, which we interpret as measuring how much information is generated by the particular outcome from the repertoire of possible outcomes (possible according to distribution *p*). It follows that

$$H\left(p\right)=E_{p}\left\{H\left[\delta_{i}\text{ }\left\|\text{ }p\right.\right]\right\}=\sum_{i} p_{i}\cdot H\left[\delta_{i}\text{ }\left\|\text{ }p\right.\right],$$

so entropy is the *expected* information generated by an outcome of repertoire *p*. Thus entropy can be derived from the more fundamental notion of relative entropy.

## *2.* The maximum entropy distribution on a finite set

The maximum entropy (maxent) distribution on a finite set *Z* of possible outcomes is the distribution *m* that maximizes the entropy functional: $m\left(Z\right):=\mathrm{argmax}_{p}H\left(p\right)$. If there are *N* outcomes in *Z* then the maxent distribution is given by

$$m\left(z_{i}\right)=\frac{1}{N}\text{ for all }z_{i}\in Z.$$

The maxent distribution on *Z* coincides with the uniform distribution on *Z*. In the paper, the set *Z* will arise as the set of possible outputs of a collection of *n* elements in a system *X*. If the elements are all binary (with outputs 0 and 1), then *Z* consists of $N=2^{n}$ possible activity patterns. A useful property of the maxent distribution is that it is independent on disjoint subsets of the system.

## *3.* The *a posteriori* repertoire

Suppose we have a subset *S* of a discrete system *X*. We detail the computation of the *a posteriori* repertoire $p\left(S_{0}\left|s_{1}\right.\right)$ specified when *S* transitions to $s_{1}$. The approach is completely general: it applies to the entire system *X*, or any part thereof.

To specify the *a posteriori* repertoire we need two pieces of data: the *a priori* repertoire of perturbations, and a description of the mechanism by which *S* responds to perturbations. The *a priori* repertoire is given by the maximum entropy distribution on the set of outputs of the elements. The mechanism is the causal architecture of the system; we suppose it to be encoded in the form of a conditional probability distribution: $p^{sys}\left(x_{1}\left|do\left(x_{0}\right)\right.\right)$, the probability the system outputs state $x_{1}$ given it is perturbed with $x_{0}$, where the $do\left(-\right)$ operator is introduced following [2]. Since we are interested in the causal interactions that are accounted for internally by *S*, we ignore the responses of elements outside of *S*, and only consider the conditional distribution $p^{sys}\left(s_{1}\left|do\left(x_{0}\right)\right.\right)$. Perturbations introduced outside of *S* can affect the response by *S*, and are not accounted for internally, so we average (marginalize) over them. Each connection afferent to *S* is averaged over using the maximum entropy distribution. After averaging out extrinsic noise, the mechanism of an element $n^{k}$ in *S* is given by:

$$p^{mech}\left(n_{1}^{k}\left|do\left(s_{0}\right)\right.\right)=\sum_{e_{0}} p^{mech}\left(n_{1}^{k}\left|do\left(s_{0},e_{0}\right)\right.\right)\cdot p^{\max}\left(e_{0}\right),$$

where the summation is over afferent connections from the environment $E:=X\backslash S$ into *S*. The environment acts as a source of extrinsic noise that loosens the mechanism within *S*. The mechanism of the system, $p^{sys}\left(s_{1}\left|do\left(s_{0}\right)\right.\right)$ is then the combined mechanism of the elements within *S*. Imposing maximum ignorance on external inputs requires perturbing connections rather than elements, since connections from the environment to *S* are a property of *S*, and the external elements themselves are not.

Note that the alternate treatment of extrinsic noise, perturbing elements rather than wires, does not qualitatively affect the results presented in the text.

The *a posteriori* repertoire can then be captured via Bayes’ rule:

$$p\left(s_{0}\to s_{1}\right):=\frac{p^{sys}\left(s_{1}\left|do\left(s_{0}\right)\right.\right)p^{\max}\left(s_{0}\right)}{p\left(s_{1}\right)},$$

where $p\left(s_{1}\right)=\sum_{s_{0}} p^{sys}\left(s_{1}\left|do\left(s_{0}\right)\right.\right)p^{\max}\left(s_{0}\right)$. Bayes’ rule provides a formal method for keeping track of the (probabilistic) effects of *a priori* perturbations introduced into the system, and for converting the resulting data into the *a posteriori* repertoire.

We consider two extreme cases. First suppose *S* is completely deterministic. It follows that

$$p\left(s_{0}\to s_{1}\right)=\left\{\begin{matrix} 1/m \text{if }p\left(s_{1}\left|do\left(s_{0}\right)\right.\right)=1 \\ 0 \text{else} \end{matrix}\right.$$

where *m* is the number of perturbations in the *a priori* repertoire that cause $s_{1}$. The *a posteriori* repertoire specified by *S* is simply all the perturbations that result in $s_{1}$, weighted equally. From *S*’s point of view the various perturbations in the *a posteriori* repertoire are indistinguishable. Note that the *a posteriori* does not specify the probability of $s_{0}$ leading to $s_{1}$ (which is 1 or 0 in this case), but rather the probability that $s_{0}$ *led* to $s_{1}$.

Second, suppose *S* operates completely randomly: each outcome is equally likely regardless of prior inputs. This may be a result of the mechanism implemented by the elements in *S*, or due to external interactions overwhelming *S*. Either way, *S* does nothing to rule out alternatives by entering $s_{1}$, and the *a posteriori* repertoire is $p\left(S_{0}\to s_{1}\right)=p^{\max}\left(S_{0}\right)$.

In an isolated deterministic system, Bayes’ rule simply keeps track of which perturbations lead to $s_{1}$, and renormalizes so that the probabilities sum to one as required. In a probabilistic system Bayes’ rule keeps track of the additional data given by the likelihoods of the perturbations given the response, and weights the perturbations according to their likelihoods.

## *4.* No integrated information is generated by a disconnected system

Suppose *X* consists of two disjoint subsets *A* and *B*. We show that $ei\left(X_{0}\to x_{1}/℘\right)=0$ for the partition $℘=\left\{A,B\right\}$, regardless of the state $x_{1}$. Recall that relative entropy is zero if and only if the two repertoires under comparison are identical, so it suffices to show that the *a posteriori* repertoire specified by the parts coincides with that specified by the system.

If *A* and *B* are disjoint then the conditional probability $p\left(x_{1}\left|x_{0}\right.\right)=p\left(a_{1},b_{1}\left|a_{0},b_{0}\right.\right)$ splits into a product: $p\left(x_{1}\left|x_{0}\right.\right)=p\left(a_{1}\left|a_{0}\right.\right)\cdot p\left(b_{1}\left|b_{0}\right.\right)$. It follows that the *a posteriori* repertoire also decomposes into a product, and so the combined *a posteriori* repertoires of the parts coincides with the *a posteriori* repertoire of the system.

## *5.* The expectation of effective information

In the course of computing the *a posteriori* repertoire given state $s_{1}$ we computed the probability of the system entering $s_{1}$ given its causal architecture and the *a priori* repertoire of perturbations. It follows that we can compute the distribution $p\left(S_{1}\right)$ of possible outcomes at $t=1$. We use $p\left(S_{1}\right)$ to compute the expected value of effective information.

First observe that effective information decomposes as a sum of simpler terms:

$$\begin{matrix} ei\left(S_{0}\to s_{1}/℘\right)=H\left[p\left(S_{0}\to s_{1}\right)\text{ }\left\|\text{ }\prod_{k} p\left(M_{0}^{k}\to\mu_{1}^{k}\right)\right.\right] \\ =\sum_{k} H\left[p\left(M_{0}^{k}\to s_{1}\right)\text{ }\left\|\text{ }p\left(M_{0}^{k}\to\mu_{1}^{k}\right)\right.\right]+H\left[p\left(S_{0}\to s_{1}\right)\text{ }\left\|\text{ }\prod_{k} p\left(M_{0}^{k}\to s_{1}\right)\right.\right]. \end{matrix}$$

The expectation of $H\left[p\left(M_{0}^{k}\to s_{1}\right)\text{ }\left\|\text{ }p\left(M_{0}^{k}\to\mu_{1}^{k}\right)\right.\right]$ is

$$\sum_{s_{1}} p\left(s_{1}\right)H\left[p\left(M_{0}^{k}\to s_{1}\right)\text{ }\left\|\text{ }p\left(M_{0}^{k}\to\mu_{1}^{k}\right)\right.\right]=H\left(p\left(M_{0}^{k}\to M_{1}^{k}\right)\right)-H\left(p\left(M_{0}^{k}\to S_{1}\right)\right)$$

and the expectation of $H\left[p\left(S_{0}\to s_{1}\right)\text{ }\left\|\text{ }\prod_{k} p\left(M_{0}^{k}\to s_{1}\right)\right.\right]$ is

$$\sum_{s_{1}} p\left(s_{1}\right)H\left[p\left(S_{0}\to s_{1}\right)\text{ }\left\|\text{ }\prod_{k} p\left(M_{0}^{k}\to s_{1}\right)\right.\right]=\sum_{k} \left[H\left(p\left(M_{0}^{k}\to S_{1}\right)\right)\right]-H\left(p\left(S_{0}\to S_{1}\right)\right).$$

In both formulae we are now computing entropy rather than relative entropy. Define the expected effective information generated by a subset *S* of *X* as

$$EI\left(S_{0}\to S_{1}\right)=H\left(p^{\max}\left(S_{0}\right)\right)-H\left(p\left(S_{0}\to S_{1}\right)\right).$$

It follows that the expectation of effective information across a partition is

$$\begin{matrix} EI\left(S_{0}\to S_{1}/℘\right):=E_{S_{1}}\left[ei\left(S_{0}\to s_{1}/℘\right)\right] \\ =EI\left(S_{0}\to S_{1}\right)-\sum_{k} \left[EI\left(M_{0}^{k}\to M_{1}^{k}\right)\right]. \end{matrix}$$

Thus the expected effective information generated across a partition is given by the expected effective information generated by the system minus (over and above) the expected effective information generated by the parts.

Finally, define *expected* integrated information, $\Phi\left(S_{1}\right)$, as expected effective information across the partition that generates the lowest quantity of normalized expected effective information:

$$\Phi\left(S_{1}\right)=EI\left(S_{0}\to S_{1}/℘^{MIP}\right).$$

The expected integrated information provides a useful characterization of the capacity of a discrete system.

## *6.* Bounds on $\Phi\left(X_{1}\right)$

Computing effective information for every partition of a set *S* is computationally intensive: the number of partitions grows faster than $e^{n}$ as a function of the number of elements *n* in the set. We show that restricting to bipartitions gives a lower bound on the expected integrated information $\Phi\left(X_{1}\right)$. Suppose we have a subset *S* of *X* and bipartition $℘=\left\{A,B\right\}$ of *S*. Notice that $EI\left(S_{0}\to S_{1}/℘\right)\geq0$ since the expression is an expectation of relative entropies, all of which are non-negative. It follows that

$$EI\left(A_{0}\to A_{1}\right)+EI\left(B_{0}\to B_{1}\right)\leq\text{ }EI\left(S_{0}\to S_{1}\right)$$

for any bipartition of *S*. This can be interpreted as saying that the whole is always (on average) at least the sum of its parts.

For any partition we can form a refinement by subdividing one or more of the parts of . Thus a bipartition can be refined to form a tri-partition, and so forth. At the bottom we find the partition into the individual elements, and below that we place the total partition, where there are no parts and the system is taken as a whole. Equation has the following consequence:

$$EI\left(X_{0}\to X_{1}/℘\right)\leq EI\left(X_{0}\to X_{1}/℘'\right)\text{ if }℘'\text{ is a refinement of }℘\text{. }$$

It follows that effective information for the minimum information bipartition provides a lower bound for $\Phi\left(X_{1}\right)$ and that effective information for the total partition provides an upper bound. An additional upper bound – for systems consisting of elements with identical outputs – is given by twice the effective information on the minimum information mid-bipartition, as can be seen by considering the normalizing constant.

***Homogeneous systems.*** We show that expected effective information in a homogeneous system of binary elements is at most one bit: $EI\left(X_{0}\to X_{1}\right)\leq1$ bit. Expanding, we see that $EI\left(X_{0}\to X_{1}\right)=H\left(p\left(X_{1}\right)\right)-H\left(p\left(X_{1}\left|X_{0}\right.\right)\right)$. The first term quantifies the repertoire of possible outputs of the system. However for a homogeneous system the elements act monolithically regardless of the input: they all either fire or all silent. Thus $H\left(X_{1}\right)\leq1$ bits. It follows from the nature of the normalization for different partitions that expected integrated information is at most one bit.

***Lattices.*** Expected effective information across the minimum bipartition in an $n\times n$ lattice of binary elements is at most $2n$ bits. There are $2n$ elements that lie on the boundary of a vertical or horizontal bipartition on an $n\times n$ lattice. These elements are the only elements that are affected by interactions between the parts, and they can at most generate 1 bit of information on average. If we assume the lattice wraps around to form a torus, as in the Game of Life grid in Figure 15, there are two cuts across which parts can interact and the upper bound increases to $4n.$

## *7.* Relationship to effective information for stationary systems

In previous work [3,4] the notions of effective information and integrated information were introduced for stationary multivariate Gaussian random process. Fix a bipartition $℘=\left\{A,B\right\}$. The effective information $EI\left(A\to B\right)=H\left(A^{\max}\right)+H\left(B\right)-H\left(A^{\max},B\right)$ across the bipartition captures the *capacity* of the subsets for integrated information. Integrated information of the system was then defined as the capacity across the minimum information bipartition; we denote it by .

In this paper we generalize effective information as a time and state dependent measure. The measure quantifies the effective information generated by the process of ruling out a collection of alternatives from the *a priori* repertoire. It cannot be directly connected to the previous notion since they apply in different contexts, however, we investigate how expected integrated information can be adapted to stationary systems since *expected* effective information can be interpreted as a capacity: the capacity for interactions between a collection of parts.

Recall that the expected value of effective information, Eq , is given by $EI\left(A_{0}\to A_{1}\right)=H\left(p^{\max}\left(A_{0}\right)\right)-H\left(p\left(A_{0}\to A_{1}\right)\right)$, and expected effective information over an arbitrary partition is $EI\left(X_{0}\to X_{1}/℘\right)=H\left(p\left(A_{0}\to A_{1}\right)\right)+H\left(p\left(B_{0}\to B_{1}\right)\right)-H\left(p\left(X_{0}\to X_{1}\right)\right)$. The most natural way to apply this to a stationary (timeless) system is to drop the time dependence and rewrite it as

the mutual information over the partition. Finding the minimum information partition, suitably normalized, and computing mutual information across the *MIP* recovers the *perturbation free* modification of integrated information discussed in [3]:

$$\Phi_{stat}^{corr}\left(X\right)=MI\left(X/℘^{MIP}\right).$$

It provides a measure of the minimal observed dependencies in a stationary system, and could be applied, for example, to determine the natural components of a stationary system.

Perturbing elements in a stationary system allows one to observe the effects one part can have on the rest of the system, but not the effects the entire system can have on itself. The perturbation free measure above detects correlations between subsets of the system, but does not detect causal interactions. This paper avoids these problems by considering non-stationary systems so that perturbations are introduced in one time step, and the response is measured in the next.

Finally, note that integration (not to be confused with integrated information) as introduced in [5] is given by $MI\left(X/\left\{X_{k}\right\}_{k=1}^{N}\right)=\sum_{k=1}^{N} H\left(X_{k}\right)-H\left(X\right)$.

## *8.* Comparison with other information measures on discrete systems, and discussion of technical aspects of $\varphi$

It is worth briefly discussing the relation between the present work, and the notions of stochastic interaction, transfer entropy and information flow.

A measure of stochastic interactions within a discrete system was introduced in [6], motivated by the desire to extend the infomax principle of [7] to dynamical processes. In their work they found that optimizing stochastic interaction results in systems with almost deterministic global behavior, and long cycles with relatively few branch points. Further, the individual elements in the optimized systems were extremely poor predictors of the system’s future behavior. Thus the optimized systems possess an intriguing mix of local flexibility and global rigidity, suggesting they may generate high $\varphi$.

Stochastic interaction is defined as follows. A discrete system *X* is given, with an initial probability distribution *p* on the outputs of the elements (in examples *p* is a stationary distribution) and causal mechanism *M*. The system is partitioned into its individual elements: $℘=\left\{\left\{n^{k}\right\}_{k=1}^{N}\right\}$, and for each element $n^{k}$ the distribution *p* is projected to its marginal distribution $p^{k}$. The causal mechanism (Markov kernel) $M^{k}$ of each part is computed in a manner similar to that in this paper. Stochastic interaction is then

$$I\left(p,M\right)=\sum_{k} H\left(p^{k},M^{k}\right)-H\left(p,M\right),$$

where $H\left(p,M\right)=\sum_{x_{0}} p\left(x_{0}\right)\cdot H\left(M\left(X_{1}\left|x_{0}\right.\right)\right)$. By comparison, *expected* effective information over and above the partition into individual elements is

$$EI\left(X_{0}\to X_{1}/\left\{\left\{n^{k}\right\}_{k=1}^{N}\right\}\right)=\sum_{k} H\left(p\left(N_{0}^{k}\to N_{1}^{k}\right)\right)-H\left(p\left(X_{0}\to X_{1}\right)\right).$$

Thus, in a special case stochastic interaction (with the maximum entropy distribution) can be seen to be similar to a special case of expected effective information (across a particular partition, i.e. the partition into individual elements). The underlying motivations however are quite different. Stochastic interaction measures the total statistical dependence introduced into the system by the action of the Markov kernel and can be thought of as a dynamic implementation of the notion of integration in [5]; whereas $\varphi$ is a measure of the repertoire of states specified by the system as a whole.

Contrasting the two measures is a useful exercise, since they seek to answer different questions using similar information-theoretic tools. We emphasize the differences, not to criticize the notion of stochastic interaction, but rather to provide a stage for motivating and summarizing certain technical aspects of the definition of $\varphi$, complementing the phenomenological presentation in the main text.

1. *Stationary versus maximum entropy distributions.* The stationary distribution(s) of a Markov process are for many purposes the most natural to use in an analysis of its dynamics, since it describes the equilibrium behavior. $\varphi$ does not capture or characterize equilibrium behavior, but is rather concerned with how a system reduces *a priori* ignorance. The maximum entropy distribution captures the notion of maximal ignorance; which is then reduced by the mechanism. It also has the important consequence that $\varphi$ is bounded, which is not true in general of relative entropy.
2. *Averaging over states versus considering a particular state.* Since we are interested in the information generated by the system, we attend to the particular state it enters, rather than take an average of states.
3. *Looking forward versus looking backwards.* Stochastic interaction quantifies how the Markov kernel transforms a distribution at $t=0$ to a distribution at $t=1$, “counting” how many different outcomes the system generates compared to its elements; i.e. by looking forward at where the system could go. In contrast, effective information quantifies the information generated by the system transitioning from $x_{0}$ to $x_{1}$ using the *a priori* and *a posteriori* repertoires; i.e. by looking back at the possible prior perturbations through the lens of the mechanism. Looking forward quantifies the number of outcomes the system can produce in general, without attending to the actual state the system is in; whereas looking back asks how much information the system generates upon entering its actual state. Consider, for example, a six-sided die. We take the position that the die does not generate information in and of itself (i.e. because it is six-sided); but rather that it must be thrown, and generates information in the act of landing on a particular face, ruling out the alternatives.

Returning to the analysis of discrete systems, as an external observer all we see is the first state followed by the second (note that $x_{0}$ may in fact be the same as $x_{1}$: the mechanism may cause the system to stay in the same state). However, the system, we have to assume, has certain undeniable properties:

- 1. at $t=1$ it is in a particular state;
  2. it has a mechanism, which admits a description as an input-output table; and
  3. it has a repertoire of possible inputs; about which the system has no *a priori* information.

It follows that there is a certain amount of effective information associated with entering any particular state. It is fruitful to visualize the mechanism as a filtering device. Each input in the *a priori* repertoire leads to a particular output, so the mechanism acts by grouping inputs into boxes labeled by outputs (possibly probabilistically). It is the action of the mechanism, taking inputs and determining the corresponding outputs, that generates information. The particular state the system is (or was) in is not informative in and of itself. Thus we are not interested in where the system was or even where it is *per se*, but rather in *how* the mechanism reduces uncertainty by entering the current state. The answer is given by the rules implemented by the elements, which we unfold by using the *a priori* repertoire to determine *how* the rules act on different possible states. Perturbing with all possible states ensures we exhaustively uncover the processing of the mechanism. By looking back at the prior perturbations through the lens of the mechanism, from the vantage point of the current state, we see how the *a priori* repertoire is contracted into the *a posteriori* repertoire by the processing of the system. Contrasting the two repertoires captures the “differences that make a difference” to the system.

As an example, consider Figure SI-1. In panel *(a)* the system is in a steady state, with no elements firing. Nevertheless, it generates 1 bit of integrated information each time it does nothing (the minimum partition in this case is the total partition). The mechanism causes the system to actively choose to remain silent. From the vantage point of the current state, the four *a posteriori* states that lead to state $x_{1}$ – which the system cannot distinguish among – are specified against the background of the eight possible *a priori* states, through the lens of the system’s mechanism. The system in panel *(b)* is also in a fixed point, with no elements firing. However, in contrast to panel *(a)*, this is not as a result of causal interactions, but rather a result of the elements ignoring their inputs and remaining silent no matter what. For this system we find that integrated information is zero bits, although to the external observer its behavior is identical to that in panel *(a)*.

1. *Difference of entropies versus directly comparing repertoires.* Stochastic interaction is the difference of the entropy generated by the elements and the entropy generated by the whole. By contrast, and crucially for our purposes, effective information directly compares the repertoires of the parts and the whole, without first converting each into a number, and then comparing the two numbers.

We quantify the information generated by the system, over and above that generated by a collection of parts, by computing the entropy of the *a posteriori* repertoire of the system relative to the combined *a posteriori* repertoires of the parts. A conceivable alternative would be to subtract the entropy of the system’s *a posteriori* repertoire from the sum of the entropies of the *a posteriori* repertoires of the parts. This would provide a measure that compares numerically the reduction in uncertainty performed by the parts with that done by the whole. Note however that we wish to capture *how* the processing of the whole differs from that of the parts: the *a posteriori* repertoire serves as a means to expose the action of the mechanism, rather than an end to be quantified in itself. It is therefore necessary to determine, for each perturbation taken separately, how the processing of the system differs from the combined action of the parts. This ensures that integrated information, as defined in the text, is zero if and only if the parts and the whole specify *identical* repertoires, which is not necessarily the case if we simply compare the sizes of the repertoires. Thus, rather than simply compare the sizes of the repertoires as seen through the lenses of the whole and the parts, we directly contrast the distributions, perturbation by perturbation. In this way, computing relative entropy, we directly compare the processing of the system as a whole to the system taken as a collection of parts.

1. *Partitions.* Stochastic interaction is computed using the partition into individual elements. Suppose we have a system of 4 elements, decomposing into 2 separate couples with internal connections, but no connections between the couples. Stochastic interaction will be nonzero, which makes sense, since there are indeed interactions within the couples. However any measure of integration must be zero on a disjoint system, since a disjoint system is not a single entity. Thus $\varphi$ is computed by comparing effective information across all partitions.

We complete the discussion by considering the notions of transfer entropy (*TE*) and information flow *(IF*). Transfer entropy [8] can be written (for special case $l=k=1$) as

$$T_{A\to B}=\sum_{a_{0},b_{0}} p\left(a_{0},b_{0}\right)\cdot H\left[p\left(B_{1}\left|a_{0},b_{0}\right.\right)\left\|p\left(B_{1}\left|b_{0}\right.\right)\right.\right].$$

It is an average of relative entropies over some initial probability distribution. It captures how much information flows from *A* to *B* by measuring how much variability in *B* is explained by *A*. Information flow [9,10] is a similar measure capturing the flow of information between two subsets of a discrete system using causal interventions.

We do not compare effective information with these measures in detail. Instead it is illuminating ask the question: what prevents transfer entropy and information flow from forming suitable foundations for measuring integrated information? Either measure could be computed across bipartitions, using $T_{A\to B}+T_{B\to A}$ for example. Comparing across all bipartitions using a normalization, would then result in measures superficially similar to $\varphi$. This sort of approach arguably provides the most straightforward way to adapt effective information from stationary to non-stationary systems, so it is worth explaining why we did not follow this route.

To avoid repeating the presentation in the text, and the discussion of stochastic information above, we restrict attention to a single aspect of our reasoning. Integrated information is motivated by a desire to understand how a discrete system acts (generates information) as a single entity. We directly compare the processing performed by the system as a whole with the processing performed by the parts – considered as wholes in their own right – to quantify the interactions in the system that *cannot be reduced* to the actions of independent subsystems. In contrast, *TE* and *IF* decompose a system into parts from the start: they quantify the flow of information (suitably defined) from one part, *A*, of a system to another, *B*. Thus, *TE* and *IF* take a fundamentally local perspective on the system that precludes understanding it as a single entity.

A simple example illustrates the point. Consider the parity system of Figure 14. The system behaves as a single giant element, and generates 1 bit of integrated information. However if we try to measure integrated information using *TE* or *IF*, splitting the system into two parts, we find that each part is capable of making one bit’s worth of difference to the other, resulting in two bits of integrated information. By focusing on the effects of the parts on each other, rather than that of the system on itself (relative to the parts), we obtain an incorrect measurement. More generally, suppose we calculate the *TE* or *IF* between all the subsets of a system. It is not clear how this data can be combined to generate a single coherent picture of the processing performed by the system. Our solution is to emphasize the action of the whole over the parts, rather than the action of the parts on each other.

Note that we could not do this without introducing time as a variable into the systems. Thus previous work [3] necessarily considered the actions of parts on each other, rather than the whole relative to the parts, since (as far as we know) this is the only way to analyze causal interactions in stationary systems.

## 9. Applications of $\varphi$ and approximations

We briefly discuss possible approaches to applying $\varphi$ to neural systems. There are at least two major hurdles. The most serious is that the causal architecture of a system must be known before $\varphi$ can be measured. A further daunting problem is dealing with the combinatorial explosion in the number of partitions and states that have to be considered in large systems.

The computational burden can be reduced in a number of ways. First, the discussion of expected effective information and $\Phi$ above shows that on average it is sufficient to restrict attention to bipartitions. Second, it is often the case that most bipartitions can be eliminated as possibilities out of hand in a system that is well-studied and understood, recall the grid for example. Third, the computation of the *a posteriori* repertoires can potentially be short-circuited. For example, perturbations that involve an abnormally high or low firing rate can be dismissed. Finally, the system in question can be considered over longer time frames and approximated as a stationary system, so that the mutual information can be used instead of effective information, see [4].

Although these approximations may prove useful and interesting, a more basic point should be made. According to the theory, a neural (or any other) system is a single entity insofar as it generates a large amount of integrated information. A practical way of addressing this issue is to consider the two notions (integration and information) separately. Approximate measures of integration can be developed; for example based on functional clustering [11], or using the mutual information approximation to integrated information, or possibly by adapting transfer entropy or information flow. Although none of these measures fully captures the notion of integration, they provide useful tools in the absence of a detailed understanding of the causal mechanisms underlying mammalian brains. Once integration has been approximately established, further measures such as coherence dimension or stochastic interaction could be applied to quantify the degrees of freedom available to the system considered as a single entity, providing an approximate measure of the integrated information generated.

## *10.* Integrated information and the choice of elements and variables

We have seen in the section on complexes that it is important to properly choose the elementary components of a system. An instructive example is given by the deterministic Hopfield-type network of [12]. It has connection weights from the *j^th^* element to the *i^th^* element given by $2^{j}$, so that network activity is updated according to

$$x_{i}\left(t\right)=\sum_{j=1}^{n} 2^{j}f\left(x_{j}\left(t-1\right)\right)\text{ for }f\left(x\right)=\left\{\begin{matrix} -1 \text{if }x<0 \\ +1 \text{if x}\geq\text{0,} \end{matrix}\right.$$

where $x_{i}\left(t\right)$ describes the state of the *i^th^* neuron at time *t*. Computing integrated information they find that $\Phi=n$ bits. They then go on to observe that the network can be rewritten in the form

$$v_{i}\left(t\right)=f\left(\sum_{j=1}^{n} 2^{j}v_{j}(t-1)\right),$$

where $v_{i}\left(t\right)=f\left(x_{i}\left(t\right)\right)$ is a binary variable describing the *output* of the *i^th^* neuron, in which case $\Phi=2$ bits. The claim is that there is a family of systems with simple dynamics and interactions between elements, and with arbitrarily high $\Phi$. Moreover the discrepancy between the $\Phi$ values in the two realizations of the system would show that $\Phi$ is sensitive to a change of variables since the two systems “produce identical behavior”.

The example is a discrete system, though their adaptation of $\Phi$ and effective information from [3] differs from the one provided here. Nevertheless, the error in the example rests on the treatment of causal interactions, rather than in the implementation of $\Phi$.

Let us first consider the claim that it is possible to construct simple systems with arbitrarily high values of $\Phi$. The possible states of a single neuron are never explicitly described in [12], except to say the states are integer valued. By considering all possible $2^{n}$ initial conditions for the network, we see that each neuron has at least $N=2^{n}$ internal states. In particular each neuron stores at least *n* bits. Since $\Phi=n$ it is apparent that the network is integrating no more information than the individual neurons. The difficulty involved in constructing a system with high $\Phi$ has been shifted onto the shoulders of the neurons: it is not clear how to build neurons that are integrated objects with repertoires of $2^{n}$ for arbitrarily large *n*. This relates to the discussion of Figure 7*ab* above. If we take the units to be single objects then it is easy to construct systems with high $\Phi$ by coupling them. The problem is that the units are not integrated.

Second, consider the claim that $\Phi$ depends on the choice of variables. Is the natural choice the $2^{n}$ internal states or the 2 outputs of the elements? Since $\Phi$ measures *causal* interactions internal states are only relevant to the extent that they contribute to outputs; beyond that they are not used by the model and are in fact somewhat artificial. Once we – or rather the elements – have identified whether the internal states are positive or negative they have no further causal consequences. The difference in $\Phi$ obtained in the two computations is not a consequence of changing variables, but rather a result of measuring things that are not effectively there. $\Phi$ is 2 bits regardless of the number of elements and connections, reflecting the simple causal architecture.

## *11.* Integrated information for continuous systems

The theory developed in the paper assumes that elements have a finite repertoire of outputs, and that time passes in discrete instants. We do not develop the theory for continuous systems; indeed, it is unclear whether continuous variables exist in nature. However, it is suggested in [12] that $\varphi$ can be infinite and depends on the choice of scale for systems with continuous variables, so we briefly discuss these issues.

Relative entropy for *continuous* random variables (which have a probability density function) is

$$H\left[p\left\|q\right.\right]=\int_{X} \log_{2}\left(\frac{p}{q}\right)dp,$$

where *q* is the prior and *p* the posterior. Relative entropy, applied to measure effective information, is easily seen to be finite (briefly, the support of *p* is contained in that of *q* since *p* is specified using more data than *q*). Relative entropy is also invariant under rescaling: if we change the units of measurement, rescaling from $X=\left[0,T\right]$ to $X=\left[0,\alpha\cdot T\right]$ for example, then the scaling factor affects both *p* and *q* and cancels out. Thus systems with continuous variables pose no theoretical difficulties.

A related question is whether continuous variables provide a cheap method for constructing systems with high $\varphi$. It is possible to imagine systems (whether discrete or continuous) of coupled components with large repertoires that have high $\varphi$. As a practical matter, the question is always how such a system could be implemented. The world appears, at the smallest scales, to be comprised of discrete components with limited repertoires of available states. Thus any object *X* with a large repertoire must contain a correspondingly large number of components. However, as discussed in the section on Complexes, see Figure 7, in that case two possibilities obtain. If the elements within an object are independent then $\varphi$ will be zero, On the other hand, to the extent the elements within an object are coupled, the *a posteriori* repertoire of the object will shrink, so effective information between objects is reduced. Finally, it is also important to note that, since $\varphi$ measures causal interactions between interacting elements, one should be careful to identify variables that reflect causal states *within* a system. Specifically, macroscopic variables such as phase or position, which are typically identified by an external observer, are only relevant insofar as they correspond to intrinsic properties of the elements of a complex that make a difference to their interactions.

## *12.* Boolean functions in scrambled counting network

Each element in the scrambled counting network implements a Boolean function, which we detail. For each element we list the 8 firing patterns that cause it to remain silent. It will fire when it receives any of the remaining 8 inputs.

| Element 1 | 0000 | 0001 | 0101 | 0110 | 1000 | 1011 | 1110 | 1111 |
| --- | --- | --- | --- | --- | --- | --- | --- | --- |
| Element 2 | 0000 | 0001 | 0011 | 0111 | 1010 | 1011 | 1100 | 1111 |
| Element 3 | 0000 | 0001 | 0101 | 0110 | 1001 | 1010 | 1101 | 1111 |
| Element 4 | 0001 | 0010 | 0110 | 0111 | 1001 | 1011 | 1100 | 1110 |

## 13. Connection matrix of network in Figure 18

Row 1 shows efferent connections from element 1 to elements 1,2,3 and so forth. Blank entries signal there is no connection.

|  | .875 | -.375 |  |  | -.875 | .75 |  |
| --- | --- | --- | --- | --- | --- | --- | --- |
| .5 |  | -.375 | 1 | .875 | -.125 | .5 | .5 |
| -.25 | -.875 |  | .125 | .125 |  | .5 |  |
| -.75 | -.375 |  |  | -.125 |  | -.75 |  |
| -.625 |  | -.875 |  |  | -1 | 1 |  |
|  | -.625 | .75 |  | -.375 |  | -.5 |  |
| -.875 |  | .5 |  | -.125 | -.5 |  | -.25 |
| -.75 | -.25 |  | -.375 |  | .25 | -.875 |  |

# References

1. Cover TM, Thomas JA (2006) Elements of Information Theory. Hoboken, New Jersey: John Wiley & Sons, Inc.

2. Pearl J (2000) Causality: models, reasoning and inference: Cambridge University Press.

3. Tononi G, Sporns O (2003) Measuring information integration. BMC Neurosci 4: 31.

4. Tononi G (2004) An information integration theory of consciousness. BMC Neurosci 5: 42.

5. Tononi G, Sporns O, Edelman GM (1994) A measure for brain complexity: relating functional segregation and integration in the nervous system. Proc Natl Acad Sci U S A 91: 5033-5037.

6. Ay N, Wennekers T (2003) Dynamical properties of strongly interacting Markov chains. Neural Netw 16: 1483-1497.

7. Linsker R (1986) From basic network principles to neural architecture. Proc Natl Acad Sci U S A 83: 7508-7512.

8. Schreiber T (2000) Measuring Information Transfer. Phys Rev Lett 85: 461-464.

9. Ay N, Krakauer DC (2007) Geometric robustness theory and biological networks. Theory Biosci 125: 93-121.

10. Ay N, Polani D (2008) Information Flows in Causal Networks. Adv Complex Syst 11: 17-41.

11. Tononi G, McIntosh AR, Russell DP, Edelman GM (1998) Functional clustering: identifying strongly interactive brain regions in neuroimaging data. Neuroimage 7: 133-149.

12. Seth AK, Izhikevich E, Reeke G, Edelman GM (2006) Theories and measures of consciousness: an extended framework. Proc Natl Acad Sci U S A 103: 10799-10804.
